# Increasing neurogenesis refines hippocampal activity rejuvenating navigational learning strategies and contextual memory throughout life

**DOI:** 10.1038/s41467-019-14026-z

**Published:** 2020-01-09

**Authors:** Gabriel Berdugo-Vega, Gonzalo Arias-Gil, Adrian López-Fernández, Benedetta Artegiani, Joanna M. Wasielewska, Chi-Chieh Lee, Michael T. Lippert, Gerd Kempermann, Kentaroh Takagaki, Federico Calegari

**Affiliations:** 1 CRTD—Center for Regenerative Therapies TU Dresden, Technische Universität Dresden, Fetscherstrasse 105, 01307 Dresden, Germany; 2 Leibniz Institute for Neurobiology, Magdeburg, Brenneckestr. 6, 39118 Magdeburg, Germany; 3 German Center for Neurodegenerative Diseases (DZNE), Tatzberg 41, 01307 Dresden, Germany; 4 Laboratory in Sensory Physiology, Institute of Biology, Otto-von-Guericke University Magdeburg, Leipziger Strasse 44, 39120 Magdeburg, Germany; 5 Present Address: Hubrecht Institute, Developmental Biology and Stem Cell Research, Uppsalalaan 8, 3584 CT Utrecht, The Netherlands

**Keywords:** Cognitive ageing, Hippocampus, Adult neurogenesis, Neural stem cells

## Abstract

Functional plasticity of the brain decreases during ageing causing marked deficits in contextual learning, allocentric navigation and episodic memory. Adult neurogenesis is a prime example of hippocampal plasticity promoting the contextualisation of information and dramatically decreases during ageing. We found that a genetically-driven expansion of neural stem cells by overexpression of the cell cycle regulators Cdk4/cyclinD1 compensated the age-related decline in neurogenesis. This triggered an overall inhibitory effect on the trisynaptic hippocampal circuit resulting in a changed profile of CA1 sharp-wave ripples known to underlie memory consolidation. Most importantly, increased neurogenesis rescued the age-related switch from hippocampal to striatal learning strategies by rescuing allocentric navigation and contextual memory. Our study demonstrates that critical aspects of hippocampal function can be reversed in old age, or compensated throughout life, by exploiting the brain’s endogenous reserve of neural stem cells.

## Abstract (web summary)

Ageing affects several brain areas causing a decrease in cognitive abilities and memory. We find that increasing the endogenous potential of the hippocampus to generate new neurons throughout life rejuvenates learning and memory, indicating that neural reserves can be exploited during ageing to compensate for age- or disease-related cognitive impairments.

## Introduction

Contextual learning, spatial navigation and episodic memory are complex cognitive processes involving hippocampal function^1–3^ and, to some extent, adult neurogenesis^4–7^. Ageing severely affects these, and other, cognitive functions^8–11^ and the identification of the causes underlying these deficits and approaches to rescue them has become crucial to address the challenges of a rapidly growing ageing population. Although it has been postulated that neural reserves can be built up throughout life, or exploited in old age, to compensate for age- or disease-related cognitive impairments^12^, the true nature and potential of such reserves remain elusive.

Navigation provides a particularly clear example of age-related hippocampal impairment subject of many studies. In this context, navigational learning strategies switch during ageing from contextual (allocentric, hippocampal dependent) that rely on the cognitive representation of a map of the environment to procedural (egocentric, striatal dependent) that are based on stereotypical responses independent from spatial cues^10,13^. While both strategies can be equally effective if the target and subject positions are constant, only the former provides the flexible update of information that is essential when the target or subject change their relative locations. Occurring from rodents to humans^14,15^, this age-dependent loss in contextual navigation has been proposed to depend on several neurophysiological impairments causing, among others, a loss of afferent inputs and changes in the excitation/inhibition balance of the hippocampal circuits leading to alterations in the formation and consolidation of spatial memory representations and imbalances with the striatal, procedural memory system^8–11^.

Adult neurogenesis is a hallmark of the hippocampus and dramatically decreases with ageing^16,17^. The role of newborn neurons in the dentate gyrus (DG) is unclear but they are thought to act as novel encoding units and/or promote the sparsity of neuronal activity through feed-back inhibition onto granule cells, thus, decreasing the interference among partially overlapping contextual information^18–21^. Consistently, increasing neurogenesis in old mice improved behavioural performance and mnemonic discrimination during contextual learning^22,23^. However, unlike behavioural performance that only considers the subject’s efficacy in addressing a cognitive demand, effects of neurogenesis on the choice of learning strategies were never explored in ageing. This is important because an assessment of learning strategies, rather than their final efficiency, becomes crucial when trying to understand how the brain processes information resulting in the engagement of different memory systems. In addition, little is known on how newborn neurons contribute in refining the activity patterns beyond the DG towards downstream hippocampal areas. Whether or not an increase in neurogenesis alone is sufficient to rescue the age-dependent switch from hippocampal to striatal learning strategies and navigation was never investigated.

Therefore, given the suggested potential of adult neurogenesis to be exploited as a resource to compensate for age- or disease-related cognitive losses^4,24^, we here investigated during ageing the use of a system to genetically increase neurogenesis that was originally developed by our group in young mice^25^. Specifically, this system was based on the overexpression of Cdk4/cyclinD1 (4D for brevity) in neural stem and progenitor cells (NSC) to increase both their cell cycle activity and numbers through symmetric proliferative divisions^26,27^. This was shown to trigger the expansion specifically of long-term, symmetrically dividing, radial neural stem cells leading to an increase in the number of newborn neurons generated during embryonic development^28,29^ and in both adult neurogenic niches of young mice^25,30^.

We found that 4D was effective in compensating the physiological, age-dependent decrease in neurogenesis both acutely in old mice as well as chronically throughout life. This cell-intrinsic, genetically-driven expansion of NSC in the DG correlated with an overall inhibition of hippocampal activity that might be important in counteracting the excitation/inhibition imbalance of its downstream circuit arising during ageing. Most importantly, increased neurogenesis rescued the use of more effective contextual learning strategies and allocentric navigation, hence, rejuvenating critical aspects of brain function.

## Results

### 4D increases NSC expansion and neurogenesis throughout life

**Fig. 1:**
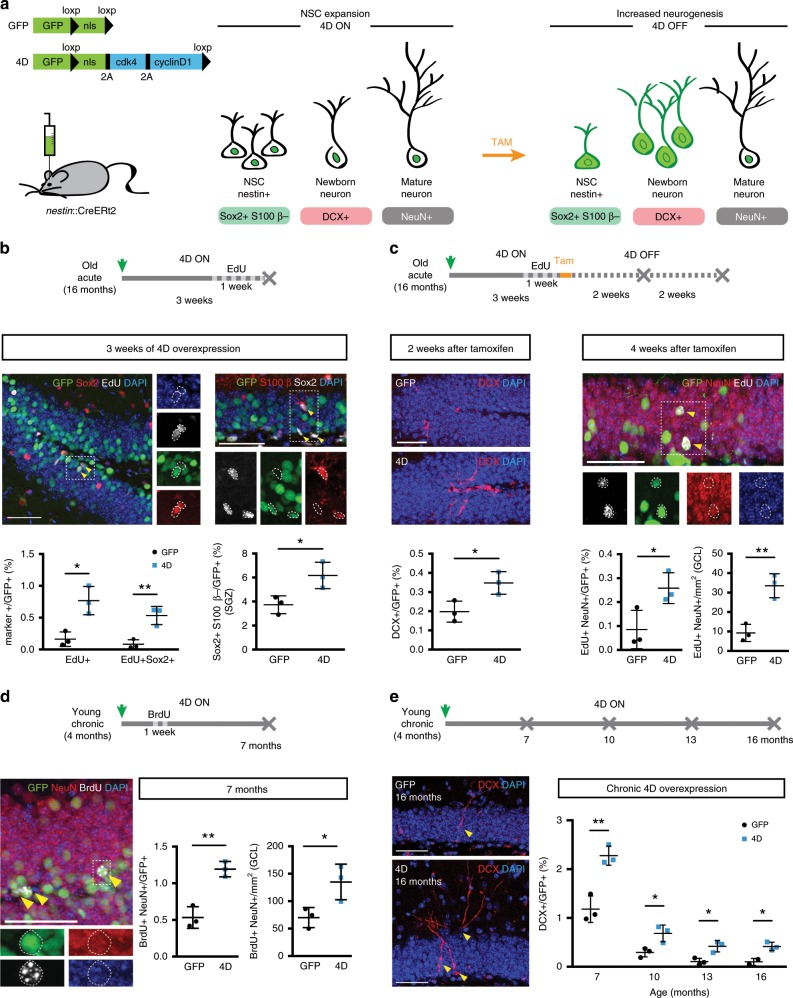
4D increases NSC expansion and neurogenesis throughout life. **a** Scheme depicting the GFP and 4D viral constructs and experimental approach to increase NSC expansion and neurogenesis. Temporal control of transgenes expression was achieved by delivering lentiviruses to *nestin*::CreERt2 mice (4D ON) with tamoxifen being later used to delete the LoxP-flanked 4D cassette (4D OFF) together with the nuclear localisation signal (nls) of GFP (or only the nls in control, GFP viruses). Effects on cell types, nuclear to cytoplasmic redistribution of GFP and neurogenic markers used in this study are depicted in the cartoon simplifying the neurogenic lineage from stem and progenitor cells (NSC) to newborn and mature neurons. **b**–**e** Experimental layouts (top), fluorescence pictures and quantifications (middle/bottom) of cell types identified upon immunohistochemistry for neurogenic markers (as indicated) in the DG or subgranular zone (SGZ) of mice injected with GFP or 4D viruses and analysed at different times later, as indicated. Insets (dashed boxes, **b**–**d**) are magnified and examples of cells scored indicated (arrowheads or dotted lines). Scale bars = 50 μm. Data represent mean ± SD as the proportion of cells scored within the GFP+ infected population or total numbers of cells per area (mm^2^) in control (black) or 4D (blue) mice; *N* = 3; *n* > 2000 (**b**–**e**) and >1000 (**b**, right); error bars = SD (except for **e**, GFP, 16 months: *N* = 2; bar = SEM). *p < 0.05; **p < 0.01 assessed by unpaired Student’s *t* test.

To investigate the potential of adult neurogenesis to rejuvenate hippocampal function, we explored the use of a system established by our group in the young hippocampus and based on 4D overexpression by stereotaxic injection of HIV-based, lentiviral vectors^25^ (Fig. 1a). Since NSC are depleted and/or lose their neurogenic potential during ageing^31,32^, it was first essential to assess whether 4D would still be effective in rescuing neurogenesis in old mice.

To this aim, 4D or GFP control lentiviruses (both carrying a nuclear-localised GFP as a reporter) were injected in the hilus of the DG of 16 months old mice. Three weeks later, the effect on the cell cycle activity of stem and progenitor cells was assessed following a 1-week thymidine labelling prior to sacrifice (Fig. 1b, top). When normalising the effect of 4D within the subpopulation of GFP+ viral-transduced cells, we found a sixfold increase in both the overall cell cycle activity (EdU+) as well as the proportion of active NSC cells (EdU+ Sox2+) relative to control mice (4D vs. GFP: 0.77 ± 0.22 vs. 0.16 ± 0.11%, unpaired Student’s *t* test *p* = 0.014 and 0.53 ± 0.14 vs. 0.08 ± 0.07%, *p* = 0.008, respectively; Fig. 1b, left). Since this increase in cell cycle activity must not necessarily correlate with a change in cell fate and a switch from neurogenic to proliferative divisions, we next assessed the 4D-effect on the size of the NSC pool (Sox2+ S100β− within the subgranular zone) finding a twofold increase in 4D relative to control mice (4D vs. GFP: 6.17 ± 1.1 vs. 3.73 ± 0.74%, unpaired *t* test *p* = 0.033; Fig. 1b, right). This doubling in the population of NSC upon 4D overexpression was essentially identical to that previously described in both neurogenic niches of young mice^25,30^ and consistent with the recent observation that activated NSC of the young and old brain react similarly to proliferative stimuli^33^.

Importantly, our use of a floxed-4D viral construct and injection in nestin-CreERt2 mice^34^ was originally designed to allow us to temporarily control the expansion of NSC by Cre recombination. In fact, our previous use of tamoxifen-dependent excision of the 4D cassette in young mice was shown to restore the physiological switch of the expanded pool of NSC to neurogenesis and resulting in the generation of an increased, age-matched cohort of newborn neurons whose maturation and integration could be followed over time^25^ (Fig. 1a). This was also confirmed in aged nestin-CreERt2 mice observing, 2 weeks after tamoxifen administration, a twofold increase in the proportion of immature neurons (Dcx+) in 4D-treated mice relative to controls (4D vs. GFP: 0.37 ± 0.06 vs. 0.19 ± 0.05%, unpaired *t* test *p* = 0.032; Fig. 1c, left). This expanded cohort of newborn neurons was found to survive and mature during the subsequent 2 weeks causing in 4D-treated mice a threefold increase in the proportion of 4 weeks old neurons birthdated prior to tamoxifen administration relative to controls (EdU+ NeuN+: 0.24 ± 0.04 vs. 0.08 ± 0.08%, respectively, unpaired *t* test *p* = 0.038; Fig. 1c, right). Notably, this relative increase in neurogenesis among the pool of GFP+, infected cells was also confirmed by a similar increase in the total number of 4-week-old neurons (EdU+ NeuN+) per area of the DG and irrespective of whether or not these neurons were derived from GFP+, infected NSC (4D vs. GFP: 34 ± 6 vs. 9 ± 4/mm^2^, unpaired *t* test *p* = 0.005; Fig. 1c, right). This in turn suggested the highly efficient targeting of nestin+ NSC and recombination by our floxed-4D viral approach. Furthermore, and highlighting the transient nature of our manipulation, 2 weeks after tamoxifen administration we observed that the pool of NSC (Sox2+ S100β−) in 4D-treated mice was restored to physiological levels (4D vs. GFP: 3.04 ± 1.04 vs. 4.18 ± 0.32, unpaired *t* test *p* = 0.142; Supplementary Fig. 1A, left) and, consistently, that the levels of newborn neurons (Dcx+) also returned to normal levels 2 additional weeks later (0.66 ± 0.40 vs. 0.58 ± 0.49%, unpaired *t* test *p* = 0.833; Supplementary Fig. 1A, right).

In addition, our approach to increase neurogenesis was designed such that the progeny of infected NSC could be morphologically identified after Cre recombination by the tamoxifen-dependent excision of the nuclear localisation signal of GFP resulting in its redistribution from the nucleus to the cytoplasm (Fig. 1a). This allowed us to assess the maturation and synaptic integration of the increased cohort of neurons by morphometric analyses at 4 and 6 weeks post-tamoxifen showing that arborisation, dendritic length and synaptic density of 4D-derived neurons were undistinguishable from those of newborn neurons equally birthdated and identified by their cytoplasmic green fluorescence upon infection of control mice with GFP viruses (4D vs. GFP: dendritic length: 0.62 ± 0.28 vs. 0.60 ± 0.23 mm, unpaired *t* test *p* = 0.795; spine density: 1.79 ± 0.31 vs. 1.89 ± 0.37/mm2, unpaired *t* test *p* = 0.326, respectively, Supplementary Fig. 1B). This was consistent with a recent report from our group in the second neurogenic niche of the subventricular zone where 4D-derived neurons in the olfactory bulb were shown to preserve their molecular, morphological and electrophysiological properties^30^.

We finally performed similar experiments in young mice to investigate whether adult neurogenesis could be persistently enhanced throughout life by a chronic 4D overexpression starting at 4 months of age and without tamoxifen treatment. Combined with neuronal birthdating as described above, this resulted in 7 months old mice (i.e. 3 months after viral injections) in both a doubling in the proportion of mature neurons (BrdU+ NeuN+) within GFP+ cells (4D vs. GFP: 1.19 ± 0.11 vs. 0.53 ± 0.15%, unpaired *t* test *p* = 0.003), as well as in their total number per area of the DG and irrespective of GFP (135 ± 31 vs. 70 ± 18/mm^2^, unpaired *t* test *p* = 0.039) (Fig. 1d). Moreover, chronic 4D overexpression starting at 4 months of age triggered a persistent increase in the generation of newborn neurons (Dcx+) in cohorts of mice analysed at 7, 10, 13 and 16 months of age ranging from two to fourfold and whose magnitude correlated with time (fold-change 4D/GFP: 2.0, 2.3, 3.7 and 4.2-fold, respectively, all unpaired *t* test *p* < 0.025; Fig. 1e and Supplementary Fig. 1C).

Together, our data showed that 4D can effectively rescue the natural decline in hippocampal neurogenesis in old age or compensate it throughout life.

### Neurogenesis enhances inhibition in the trisynaptic circuit

**Fig. 2:**
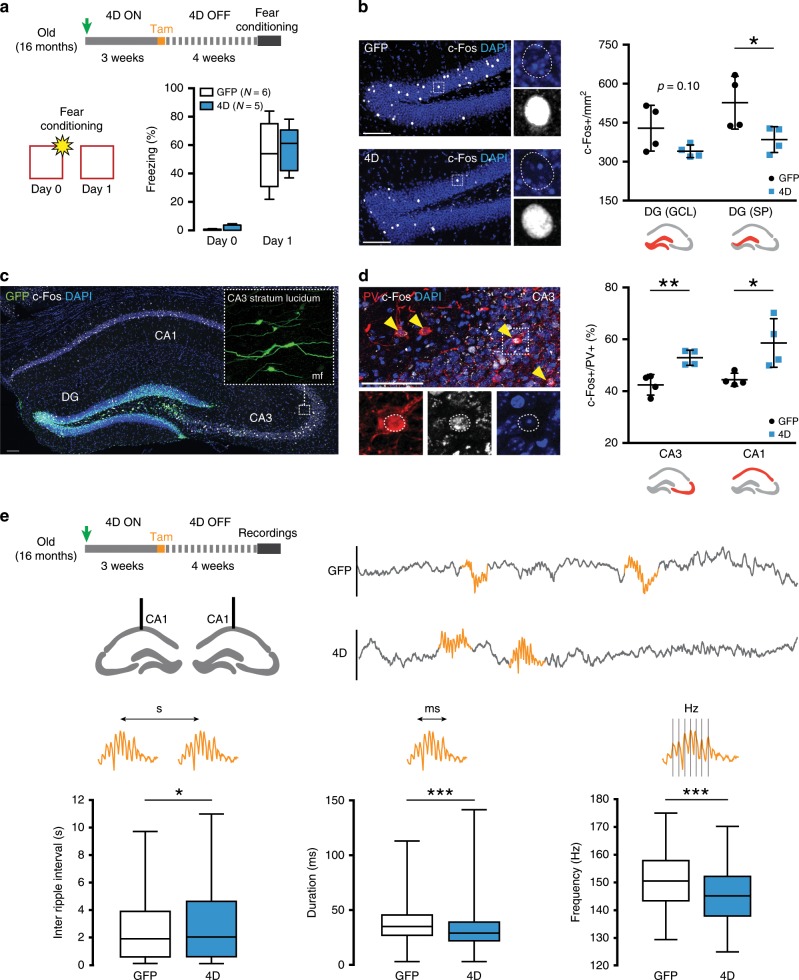
Neurogenesis decreases overall hippocampal activity of aged mice. **a** Experimental layout (top) and freezing responses (bottom) of 18 months old, GFP or 4D-treated mice subjected to a fear conditioning test 4 weeks after tamoxifen administration (as depicted) and used to assess neural activity in the trisynaptic hippocampal circuit by means of c-Fos quantification. **b**–**d** Immunohistochemistry fluorescence pictures with magnifications (left) and quantifications (right) for c-Fos in either the granule cell layer or suprapyramidal (SP) blade of the DG (**b**, as indicated) or in the Pv+ population (yellow arrowheads) of CA3 and CA1 (**d**, as indicated). Note the presence of GFP+ mossy fibres (mf) projections in the stratum lucidum of CA3. Data were expressed as c-Fos+ cells per DG area (**b**) or double positive for Pv in CA3 and CA1 (**d**, including the strata radiatum, lucidum, pyramidale and oriens, as well as the strata radiatum, pyramidale and oriens, respectively) scored in the dorsal hippocampus. Scale bars = 100 μm. **e** Experimental layout and schematic representation of brain recordings and local field potentials (top) of 18 months old GFP and 4D-treated mice used to assess sharp-wave ripples (orange lines) in natural drowsy state. Data (box–whisker plots, bottom) represent inter-ripple intervals (s), duration (ms) and internal frequencies (Hz). Note the subtle, but highly significant, changes observed in all parameters. *N* = 6 and 5 (**a**, box–whisker plots) or 4 (**b**, **d**, error bars = SD); *n* > 50 (**d**, CA3) and >10 (**d**, CA1). *N* = 4; *n* > 1500 ripples (**e**, box–whisker plots of the 10–90% quantile). *p < 0.05; **p < 0.01; ***p < 10^−10^ assessed by unpaired Student’s *t* test (**a**–**d**) and Kruskal–Wallis test (**e**).

We next investigated whether a 4D-triggered increase in neurogenesis in old mice was sufficient to alter physiological parameters of hippocampal activity thought to underlie learning and memory formation. To this end, 4D- or GFP-treated old mice were subjected to a simple fear conditioning paradigm 4 weeks after tamoxifen administration (Fig. 2a, top), i.e. when newborn neurons are known to display functional integration and enhanced synaptic plasticity^35–37^. These two cohorts of mice were then re-exposed to the same context the following day, their freezing response tested and the extent of neuronal activity in the DG assessed by c-Fos immunoreactivity as a proxy for memory representation.

First, we found that both groups of mice achieved a similar behavioural performance when re-exposed to the same context the following day with no difference in the stereotypical freezing response and indicative of a similarly efficient learning (4D vs. GFP: 57.31 ± 17.81 vs. 53.31 ± 24.57%, unpaired *t* test *p* = 0.761; Fig. 2a). However, when assessing neuronal activation in the granule cell layer of the DG during memory retrieval we found that 4D-treated mice displayed a clear trend of decreased density of c-Fos+ cells relative to controls (4D vs. GFP: 340 ± 24 vs. 429 ± 88 cells/mm^2^, unpaired *t* test *p* = 0.101) that became more prominent and significant when restricting our analysis to the suprapyramidal blade as the most active and responsive area of the DG^38^ (385 ± 50 vs. 557 ± 101 cells/mm^2^, unpaired *t* test *p* = 0.046) (Fig. 2b). While this result was consistent with the proposed role of adult neurogenesis in decreasing the activity of granule cells in the DG^39–41^, it is unclear how such neurogenesis-dependent sparsity influences the downstream activity of the hippocampus including at the level of the CA3 and CA1.

Taking advantage of the redistribution of GFP to the cytoplasm upon tamoxifen recombination, we confirmed the presence of GFP+ projections from the DG reaching the CA3 (Fig. 2c) consistent with the reported innervation of 4-week-old neurons to this area^36,42,43^. Moreover, driven by the observation of GFP+ mossy fibre terminals in the stratum lucidum of CA3 (Fig. 2c and Supplementary Fig. 2A) indicative of excitatory connections between newborn neurons and pyramidal cells, we decided to assess the number of c-Fos+ cells in this area but no major difference was found between 4D-treated and control mice neither in the CA3 nor CA1 (4D vs. GFP: 548 ± 189 vs. 692 ± 48/mm2, unpaired *t* test *p* = 0.217 and 1992 ± 1010 vs. 2429 ± 257/mm2, *p* = 0.361, respectively; Supplementary Fig. 2A). Interestingly, closer examination of these mossy fibre terminals of newborn neurons revealed characteristic filopodia (Supplementary Fig. 2B) known to contact parvalbumin (Pv) interneurons and mediate feed-forward inhibition to CA3^36,43^. Hence, we restricted our analysis of c-Fos to Pv+ cells finding that 4D increased the activity of these inhibitory interneurons in both the CA3 (4D vs. GFP: 52.91 ± 2.93 vs. 42.43 ± 3.97%, unpaired *t* test *p* = 0.005) and, surprisingly, also CA1 (58.60 ± 9.37 vs. 44.42 ± 2.51%, unpaired *t* test *p* = 0.027) (Fig. 2d). This specific increase in the activity of inhibitory interneurons in both areas, but without a corresponding change in the overall number of c-Fos+ cells in neither of the two, made us speculate that neurogenesis might alter electrophysiological parameters, such as firing patterns, that are not necessarily revealed by c-Fos immunoreactivity (supporting this, we observed that the reported hyperexcitability of the aging CA3^44^ still did not correlate with reduced c-Fos activity levels of old unmanipulated relative to young mice; Supplementary Fig. 2C). Hence, we next investigated the potential of neurogenesis in the DG to influence the basal population activity of CA1 pyramidal cells as the final destination within the trisynaptic hippocampal circuit and generating CA3-dependent activity patterns underlying memory consolidation, namely sharp-wave ripples^45–47^.

Local field potentials (LFPs) in the CA1 were recorded on cohorts of 4D and control mice during free exploration and ripples arising during natural drowsy states identified and subsequently analysed (Supplementary Fig. 2D). Remarkably, we found not only that the time between consecutive ripples increased in 4D-treated mice relative to controls (median–lower/upper quartiles, 4D vs. GFP: 2.04–0.60/4.63 vs. 1.90–0.59/3.89 s, Kruskal–Wallis *p* = 0.045) but also that this was paralleled by a reduction in both ripple duration (29–22/29 vs. 35–27/45.5 ms, Kruskal–Wallis *p* < 10^−10^) and their internal frequencies (145.16–137.93/170.21 vs. 150.54–143.39/157.89 Hz, Kruskal–Wallis *p* < 10^−10^) (Fig. 2e).

Together, our data support the role of neurogenesis in promoting sparsity in the DG through feed-back inhibition and, additionally, in regulating the activity of downstream areas through feed-forward inhibition^36,39–41^. Interestingly, this increase in the inhibitory activity of CA3 and CA1 interneurons (Fig. 2d) fit well with a concomitant reduction in the occurrence, duration and internal frequencies of sharp-wave ripples (Fig. 2e) suggesting that neurogenesis might compensate for the characteristic hyperexcitability of the old CA3 area and ultimately control critical activity patterns underlying memory consolidation^48^. Despite the absence of a clear effect in behavioural performance during a simple conditioning test (Fig. 2a), we next speculated that these differences in information processing and encoding might become relevant when challenging the mice with a difficult cognitive task that would require the adoption of one among different learning strategies.

### Neurogenesis rejuvenates navigational learning strategies

To address changes in navigational strategies during ageing, we implemented a method previously described in young mice to assess spatial learning during navigation in the Morris water maze^49,50^ and adapted this method for old mice by using a dry-land Barnes maze and including phases of learning and of re-learning after reversal (Supplementary Fig. 3A). To validate our method, we first tested navigation of young and old, unmanipulated mice finding, consistent with the known effect of ageing on navigation^8–11^, that the former group of mice displayed a greater use of contextual (allocentric) relative to procedural (egocentric) strategies than the latter throughout both the learning and reversal phases (logistic regression, odds ratio (OR) of young vs. old: contextual = 1.85 and 2.28, Wald-test *p* = 0.033 and 0.02; procedural = 0.34 and 0.48, *p* = 0.002 and 0.02, respectively; Supplementary Fig. 3A).

**Fig. 3:**
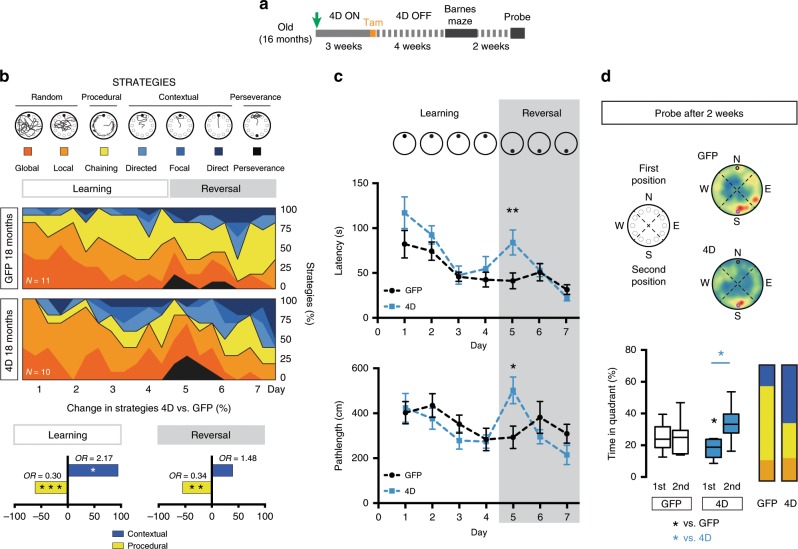
An acute increase in neurogenesis rejuvenates navigational strategies and spatial memory. **a** Experimental layout to assess the effects of acute increase in neurogenesis on navigational strategies in old mice. **b** Drawings exemplifying colour-coded searching tracks (top) and their mean relative contribution during 7 days of testing of GFP or 4D mice (as indicated) including learning and reversal phases (middle). Changes and odds ratios (OR) in contextual (blue) vs. procedural (yellow) strategies of 4D-treated relative to control, old mice are depicted by grouping together all trials of the two phases (bottom). **c** Latency (s; top) and pathlength (cm; bottom) of 4D-treated (blue) and control (black) mice performing the test shown in **b**. Note the increase in both parameters in 4D mice only the first day after reversal (day 5). **d** Schematic representation, heatmaps (top) and box–whisker plots quantifications (bottom) displaying quadrant preference of mice 2 weeks after the testing shown in **b**. Note the increased preference of 4D mice for the 2nd position of the escape box relative to both the 1st position (paired *t* test, blue *p = 0.010) and control mice (unpaired *t* test, black *p = 0.030) and the increased use of contextual vs. procedural navigation (colour coded as in **b**; bar graph, right). *N* = 11 and 10 (as indicated); values represent means of each day depicted as percentage (**b** and **d**) or numerical units (**c**). Bars in **c** SEM. *p < 0.05; **p < 0.01; ***p < 0.001 assessed by Wald-test (**b**) and paired or unpaired Student’s *t* test (for intra or inter-group comparisons, respectively; **c** and **d**).

Having validated our ability to detect differences in navigational strategies, we next investigated the effects of increasing neurogenesis in old mice treated with 4D as already described (Fig. 3a). Remarkably, while control mice almost exclusively navigated either randomly or by using procedural strategies moving in circles at the periphery of the maze (chaining), mice with acutely increased levels of neurogenesis showed a doubling in the use of contextual strategies during learning that occurred at the expense of procedural ones (OR 4D vs. GFP: contextual = 2.17; procedural = 0.30, Wald-test *p* = 0.038 and 0.001, respectively; Fig. 3b and Supplementary Fig. 3B). Moreover, even after reversal 4D-treated mice continued to develop allocentric navigational strategies more efficiently than controls leading, in the last day of testing, to a doubling in the use of contextual, and reduced procedural, navigation (OR 4D vs. GFP: contextual = 3.25; procedural = 0.30, Wald-test *p* = 0.036 and 0.027, respectively; Fig. 3b and Supplementary Fig. 3B).

These experiments gave us the opportunity to also analyse standard parameters traditionally used to assess behavioural performance such as latency and pathlength. Surprisingly, while during learning both latencies and pathlengths showed a clear improvement over time, neither of them showed any difference between the two groups of 4D and control mice (two-way ANOVA, latency: time F_(3,57)_ = 13.43, *p* < 0.0001, group F_(1,19)_ = 2.278, *p* = 0.147, interaction F_(3,57)_ = 0.928, *p* = 0.433; pathlength: time F_(3,57)_ = 5.560, *p* *=* 0.002, group F_(1,19)_ = 0.428, *p* = 0.521, interaction F_(3,57)_ = 0.610, *p* = 0.611; Fig. 3c). In contrast, differences were observed in the development of both parameters as the two groups of mice progressed in their re-learning phase after reversal (latency: time F_(2,38)_ = 8.839, *p* = 0.001, group F_(1,19)_ = 2.904, *p* = 0.105, interaction F_(2,38)_ = 4.892, *p* = 0.013; pathlength: time F_(2,38)_ = 3.429, *p* *=* 0.043, group F_(1,19)_ = 0.051, *p* = 0.824, interaction F_(2,38)_ = 5.55, *p* = 0.008; Fig. 3c). Intriguingly, this was due to a transient approximately twofold increase in both latency and pathlength in 4D-treated mice relative to control during the first day of re-learning (unpaired *t* test *p* < 0.016; Fig. 3c). In fact, this behaviour of 4D-treated mice was due to an increased perseverance at the previous position of the escape box relative to controls (time and crossing: unpaired *t* test *p* = 0.053 and 0.010, respectively; Supplementary Fig. 3C) and in turn explained as a consequence of an increased use of a place-directed, contextual navigation during learning (Fig. 3b). In contrast, the use of a procedural strategy by control mice, and their predominant chaining in circles at the periphery of the maze, was clearly not influenced by a switch in the goal position minimising the appearance of a perseverance behaviour and the need to re-learn. While the behavioural performance of control mice did not change until the end of testing, 4D mice completely compensated their starting deficit by the second day after reversal reaching similar, if not better, performances in the third day (Fig. 3c) and highlighting the ability of 4D mice to flexibly re-learn. Together, these data point out not only the potential of adult neurogenesis to rescue the age-related decrease in allocentric navigation and flexible learning but also the importance of interpreting changes in behavioural performance only within the contexts of defined cognitive processes.

A probe trial was performed on the same two groups of mice 2 weeks later finding that control mice had no preference for neither the learning nor the reversal position of the escape box (time in first vs. second position quadrant: 24.8 ± 2.3 vs. 25.0 ± 3.0%, paired *t* test *p* = 0.955; Fig. 3d) and continued to display procedural behaviour by navigating at the periphery of the maze as before. In contrast, 4D-treated mice displayed a twofold increased preference for the reversal position (first vs. second position: 17.9 ± 1.8 vs. 33.5 ± 3.3%, paired *t* test *p* = 0.011; Fig. 3d) showing that a rescue in allocentric navigation upon increased neurogenesis in old mice was also associated with the better acquisition of a place memory that was absent in controls.

We next sought to extend our observations to a behavioural paradigm not involving navigation. To this aim, we established a contextual discrimination test with an alternation in the context presentation each subsequent day (Supplementary Fig. 4A). This was used as a means to test whether mice distinguished the fear-conditioned context A relative to a similar non-conditioned context B irrespective of the presentation order as opposed to freezing in whatever context was presented first and reminiscent of contextual vs. procedural behaviour, respectively. We first assessed the validity of this behavioural paradigm testing young and old unmanipulated mice, both of which initially showed a similar degree of freezing in both context A and B indicating a comparable generalisation and behavioural performance during training (Supplementary Fig. 4B). After this training phase, young mice showed an increasing level of discrimination (more freezing in A than in B) in all consecutive days with no difference between days, in which the presentation order was reversed (A-B vs. B-A days: 0.26 ± 0.16 vs. 0.39 ± 0.17, paired *t* test *p* = 0.605) indicative of successful contextual learning. In contrast, old mice showed positive levels of discrimination only in days with a A-B but not B-A order (0.14 ± 0.06 vs. −0.03 ± 0.07, paired *t* test *p* = 0.018; Supplementary Fig. 4C) suggesting impaired contextual, and reminiscent of procedural, learning.

When testing 4D and control old mice, we found not only that mice with increased neurogenesis showed reduced levels of generalisation relative to control during the training phase (freezing in B, 4D vs. GFP: 59.94 ± 12.31 vs. 70.51 ± 2.53%, unpaired *t* test *p* = 0.032; Supplementary Fig. 4D, left) but also that during the testing phase in the following days these mice displayed a reduced and less significant intragroup bias for the presentation order (A-B vs. B-A: 0.10 ± 0.05 vs. −0.02 ± 0.07, paired *t* test *p* = 0.101) than that observed in control mice (0.19 ± 0.08 vs. 0.00 ± 0.07, paired *t* test *p* = 0.013) (Supplementary Fig. 4D, right). Yet, despite these relatively minor improvements, 4D-treated mice did not show any apparent increase in discrimination in B-A days (Supplementary Fig. 4D, right) suggesting that despite increased neurogenesis this task remained too difficult for such an advanced age. Since mice must have lost contextual discrimination between youth and senescence, we speculated that this impairment could be delayed by a chronic expansion of NSC throughout life.

### Neurogenesis preserves hippocampal learning throughout life

**Fig. 4:**
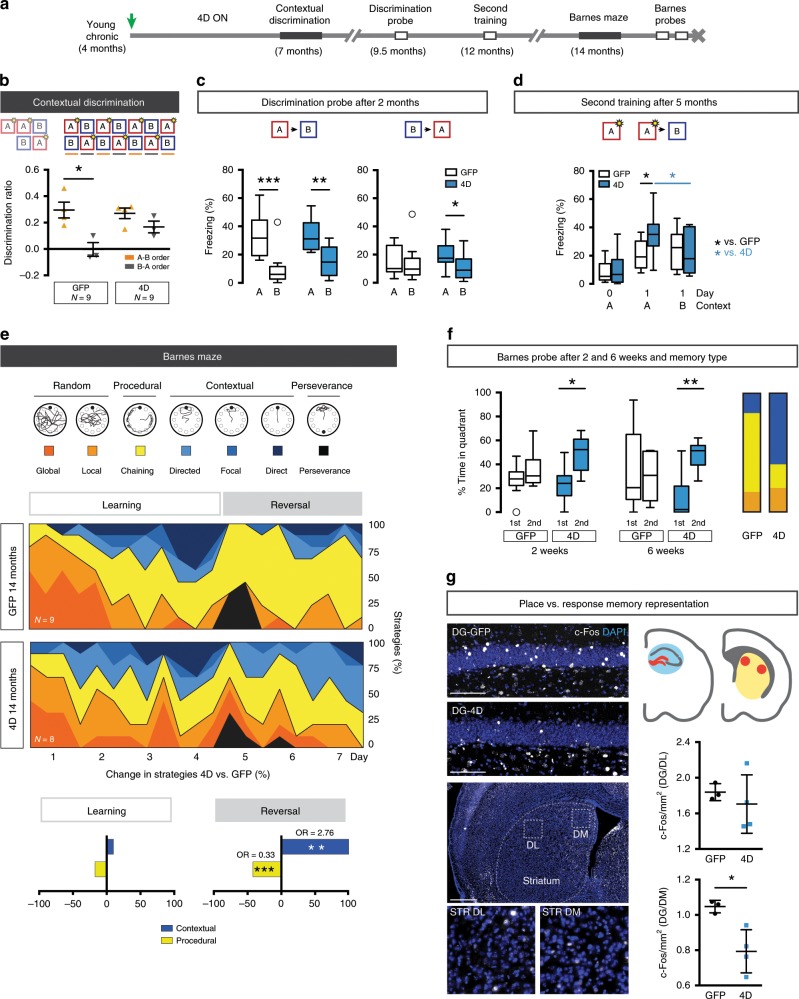
A chronic increase in neurogenesis preserves hippocampal learning throughout life. **a** Experimental layout of mice injected with GFP or 4D viruses at 4 months of age (green arrow) and tested for over 1 year (as indicated). **b**–**d** Freezing response during a contextual discrimination test performed by altering the presentation order of a context A and B each day for 7 consecutive days (as depicted in **b** and in Supplementary Fig. 4A), with ratios for days with A-B (orange) and B-A (grey) order during the discrimination phase (**b**) and box–whisker plot quantifications of relative freezing response (%) during a probe trial (**c**) and second training phase (**d**) performed different months later (as indicated) for GFP and 4D chronically treated mice. Note the positive discrimination ratio of 4D-treated mice independent from the presentation order during both testing (**b**) and probe trial (**c**) and the more efficient reacquisition of memory after a second training (**d**). **e**, **f** Assessment of the relative contributions of navigational strategies of 14 months old mice (i.e. 2 months after tests shown in **d**) (**e**) and box–whisker plot quantification of quadrant preference during probe trials performed 2 and 6 weeks later (**f**). Note the improvements of 4D-treated mice during flexible re-learning after reversal (**e**; bottom) and the preservation of a space memory absent in controls (**f**; right). **g** Fluorescence pictures of the DG and striatum (left) of mice tested for remote memory (**f**) stained for c-Fos and DAPI (as indicated). Insets (bottom) indicate the dorso-lateral or medial (DL or DM, respectively) striatal area within which quantifications were performed (right). Data represent activation ratios extracted from total c-Fos+ cells per area (mm^2^) (Supplementary Fig. 4E) in the respective brain structures, as indicated. Scale bar = 100 μm (DG) and 500 μm (striatum). *N* = 9 (**b**–**d**) or 9 and 8 (**e** and **f**); *n* = 4 and 3 (**b**, representing days with A-B or B-A presentation order, respectively); *N* = 3 and 4 (**g**). Error bars = SD (**b** and **g**). *p < 0.05; **p < 0.01; ***p < 0.001 assessed as paired or unpaired Student’s *t* test (for intra or inter-group comparisons, respectively; **b**–**d** and **f**, **g**) and Wald-test (**e**).

Mice at 4 months of age were chronically treated as previously described and tested at 7 months of age (Fig. 4a). Remarkably, we found that contextual discrimination of control mice was already impaired with values similar to those of old mice and positive discrimination only in days with the A-B but not B-A order (0.30 ± 0.12 vs. 0.00 ± 0.09, paired *t* test *p* = 0.014, respectively; compare Fig. 4b and Supplementary Fig. 4D, right). In contrast, chronically treated 4D animals preserved positive discrimination ratios in consecutive days throughout the test independently from presentation order (A-B vs. B-A: 0.27 ± 0.08 vs. 0.16 ± 0.08, paired *t* test *p* = 0.144; Fig. 4b) indicative of contextual learning. To further corroborate this, the same 4D and control mice were tested 2 months later at 9 months of age on a probe trial for memory retrieval, in which both cohorts showed similar levels of discrimination in the A-B order, whereas only 4D mice discriminated in the B-A order (4D freezing in B vs. A: 10.15 ± 9.57 vs. 18.83 ± 10.00%, paired *t* test *p* = 0.051; Fig. 4c) and consistent with a retention of contextual memory in 4D mice. One additional test for memory reacquisition was performed 3 months later by a second training at 12 months of age, in which mice showed a similarly low freezing response when re-exposed to the context A. While both groups increased freezing in the next day, control mice lacked discrimination and performed similarly in both contexts (A vs. B: 20.3 ± 10.1 vs. 23.3 ± 14.1%, paired *t* test *p* = 0.619; Fig. 4d). In contrast, 4D-treated mice showed a higher contextual discrimination not only in A relative to B (35.5 ± 15.7 vs. 22.2 ± 15.8%, respectively, paired *t* test *p* = 0.030) but also relative to controls (unpaired *t* test *p* = 0.029) (Fig. 4d). Together with our previous results, these data indicated that a chronic increase in neurogenesis delayed the loss in contextual discrimination during ageing and that this was associated with a better retention and recovery of memory.

One additional aspect emerging from our study was that an increase in adult neurogenesis was associated with different behavioural gains depending on the difficulty of the task and the age of the animals. Therefore, we decided to take advantage of these life-long, chronic manipulations by testing the same mice for navigational strategies at a slightly earlier age than assessed above. At 14 months of age, control mice still preserved a relatively high degree of contextual navigation during learning that was however almost completely replaced by procedural navigation after reversal (Fig. 4e) implying that the most evident impairment in navigation at this age was primarily related to the flexibility of re-learning. Notably, a chronic increase in neurogenesis throughout life specifically compensated this effect by favouring the use of contextual strategies at the expense of procedural ones during re-learning after reversal (OR 4D vs. GFP: contextual = 2.76; procedural = 0.33, Wald-test *p* = 0.008 and 0.001, respectively; Fig. 4e).

When tested with a recent and remote memory trial 2 and 6 weeks later both control and 4D mice preserved their procedural (response) and contextual (place) memories, respectively, while only the latter showed a greater spatial preference for the last position of the escape box (ca. 2 and fourfold increase at 2 and 6 weeks, paired *t* test *p* = 0.022 and 0.003, respectively; Fig. 4f). Knowing that procedural and contextual navigation are based on a competition between the hippocampal and striatal memory systems^14,15,51^, we next assessed neuronal activity in both areas during memory expression. C-Fos+ cells were scored after the last probe test not only in the DG but also in the lateral or medial component of the dorsal striatum, as two key regions underlying procedural memory^52^. When total number of c-Fos+ cells were scored, no significant difference could be found between control and 4D-treated mice (Supplementary Fig. 4E). Interestingly however when calculating the relative activation between DG and striatal areas, we found a decreased ratio specifically in relationship to the dorsomedial striatal region (4D vs. GFP: 1.04 ± 0.03 vs. 0.79 ± 0.12, unpaired *t* test *p* = 0.019; Fig. 4g) showing a correlate to our previous observation of reduced hippocampal activity upon increased neurogenesis (Fig. 2) and differential activation of memory-related areas dedicated to allocentric vs. egocentric behaviour.

## Discussion

Here, we increased adult hippocampal neurogenesis by a cell-intrinsic genetic manipulation in NSC without systemic side effects or interfering with the physiology of the neurons themselves. This manipulation was sufficient to compensate several age-dependent deficits in hippocampal function and promote contextual learning, allocentric navigation and memory in old age or throughout life.

Extending previous studies, our work emphasises the importance of adult neurogenesis in strategy choice rather than only the efficacy of the strategies themselves. This is important because parameters used to assess behavioural performance or mnemonic discrimination, such as latency or freezing, may alone be insufficient to dissect more subtle aspects of brain function and may also depend on factors that are independent from the intrinsic computational properties of the hippocampus^53^. This was the case when mice with increased neurogenesis using allocentric navigation during learning showed a transient decrease in performance after reversal. Analysis of learning strategies not only provided a better basis for understanding effects on behavioural performance, but it also underscored the cognitive process (hippocampal vs. striatal memory system) that was engaged in the process of learning and predictive of the type of memory that will be formed (place vs. response, respectively).

We found that increasing neurogenesis promoted the sparsity of neuronal activity in the DG and for the first time report its effects on downstream hippocampal areas, their patterns of activity and relationship with the striatum during ageing. Specifically, our manipulation promoted the activity of CA3 and CA1 inhibitory interneurons and decreased the occurrence, duration and internal frequency of sharp-wave ripples as a distinctive biomarker of memory consolidation in the context of navigation^45,46^. Several observations link our study to recent reports, together highlighting a major role of adult neurogenesis in setting the tone of the hippocampus and re-engage it in the process of learning during ageing.

Specifically, the reduced activity of granule cells in the DG of 4D-treated mice is not only consistent with the suggested role of newborn neurons in triggering feed-back inhibition^36,39–41^ but can also be explained by the recent observation of monosynaptic inhibitory input from newborn neurons onto granule cells^54^. In addition, we found that an increase in newborn neurons in the DG triggered feed-forward inhibition onto CA3. While a modulatory role of DG mature granule cells in the activation of CA3 interneurons has recently been shown to decrease generalisation during ageing^55^, our observation of direct contacts between newborn neurons and parvalbumin cells, with a comparable improvement in learning and memory precision, suggests that this feed-forward inhibition can also be triggered by the newborn neurons themselves in addition to mature granule cells. Interestingly, the reduced activity of DG granule cells and increased recruitment of downstream inhibitory interneurons might be important in counteracting the known hyperexcitability of the CA3 auto-associative network that has been proposed to lead to memory rigidity during ageing^8,11^. Further, this increased inhibitory tone in the CA3 was accompanied by a decreased occurrence, duration and internal frequencies of CA1 ripples, collectively suggesting that neurogenesis might counteract the changes in excitability of the aged hippocampus^56^. Although more studies are needed to gain a mechanistic and computational understanding of these observations, our study suggests that an increase in neurogenesis alone is sufficient to refine the formation of memory traces, and their consolidation, to ultimately alter the balance between competing navigational memory systems. Notably, our study is the first to alter this competition without surgical lesions or pharmacological alterations of brain activity but solely by genetically increasing the pool of endogenous stem cells.

During ageing, impairments in contextual and spatial learning and episodic memory were suggested to depend on many complex effects ranging from general metabolism to the physiology of the brain’s circuitry and the neurons themselves^8–11^. Consistent with a previous report^57^, we observed that impairments in allocentric navigation visibly progressed from 14 to 16 and from 16 to 18 months of age, that is long after neurogenesis had already reached a minimum plateau and implying that critical factors other than neurogenesis must arise during ageing to decrease hippocampal function. Yet, and as a remarkable aspect of our study, and increase in neurogenesis alone was sufficient to compensate for these many deficits despite the fact that the number of extra-generated neurons was seemingly negligible. In fact, the drop of neurogenesis to barely detectable levels in old mice^16,17^ implied that even if we induced a twofold or even threefold increase in the relative abundance of newborn neurons upon 4D overexpression, their numbers still remained remarkably low and calculated as a total of merely ≈100 additional neurons in the whole brain. This is noteworthy first, because this seemingly insignificant increase in 4D, old mice still represented a minor fraction of those physiologically generated in young mice, yet sufficient to rescue several functional aspects of the young hippocampus. Second, because it suggested that early clinical interventions that rescue cell loss even to levels otherwise considered negligible may still provide significant functional benefits. Whether human adult neurogenesis is limited to childhood^58^, persists throughout ageing^59^ or only becomes relevant in disease^60^, our finding that an endogenous manipulation of NSC rejuvenates hippocampal function provides an important proof-of-principle to compensate or rescue cognitive impairments over the course of life.

## Methods

### Viral preparations

Lentiviruses were produced by polyethyleneimine co-transfection of 293T cells with the respective transfer vectors encoding for GFP or 4D, HIV-1 gag/pol and VSV-G as previously described^25^. In particular, 4D viruses encoded for the three transgenes (GFP^nls^/Cdk4/cyclinD1) linked by 2A peptides and with LoxP sites allowing the recombination of the 4D cassette together with the nls of GFP (Fig. 1a)^25^. Floxed-nls-GFP vectors were used for the generation of control viral particles. One day after transfection, cells were switched to serum free medium and 1 day later the filtered supernatants were centrifuged at 25.500 rpm for 4 h. The viral particles were suspended in 40 μl of PBS per 10 cm petri dish and further concentrated using centrifugal filters (Amicon) yielding ca. 40 μl of virus suspension per construct with a titre of 10^8^–10^9^ IU/ml as assessed on HEK cells. A video of this protocol is available^61^.

### Animal treatments

Animal experiments were approved by local authorities and complied with all relevant ethical regulations (24D-9168.11-1/41, 2008-16, 2011-11, TVV 39/2015 and 13/2016). Mice were kept in standard cages with a 12 h light cycle and water and food ad libitum. In all experiments, female *nestin*::CreRTt2^34^ mice in a C57BL/6j genetic background were used. Briefly, isofluorane-anaesthetised mice were stereotaxically injected with 1 μl per hemisphere of viral suspension in the hilus of the DG as previously reported^25,61^ using a nanoliter-2000 injector (World Precision Instruments) and a stereotaxic frame Model 900 (Kopf Instruments) at ±1.6 mm mediolateral, −1.9 anteriorposterior, and −1.9 mm dorsoventral from bregma with a constant flow of 200 nl/min. Recombination of the 4D cassette and nls of GFP was achieved by oral administration of tamoxifen (Sigma) dissolved in corn oil (1:10) at 500 mg/kg body weight once a day for 4 days. When appropriate, animals were intraperitoneally injected with 100 μl of BrdU (Sigma) or EdU (Sigma) dissolved in PBS at 50 or 5 mg/kg concentration, respectively. Animals were either subjected to behavioural tests and/or anesthetized with pentobarbital and transcardially perfused with saline followed by 4% paraformaldehyde fixation in phosphate buffer (PFA). Perfusions for assessment of c-Fos activity was performed 90 min after a behavioural test.

### Cellular analyses

Brains were post-fixed overnight in 4% PFA at 4 °C and cut in coronal 40 μm thick vibratome sections that were serially collected along the rostro-caudal axis of the hippocampus and stored at −20 °C in cryoprotectant solution (25% ethylene glycol and 25% glycerol in PBS). Immunohistochemistry was performed stereologically 1 every 6 sections (i.e. 10–12 sections analysed per brain) after blocking and permeabilization with 10% donkey serum in 0.3% Triton X-100 in PBS for 1.5 h at room temperature as previously described^25^. Primary and secondary antibodies (Table S1) were incubated in 3% donkey serum in 0.3% Triton X-100 in PBS overnight at 4 °C. For BrdU detection sections were exposed to HCl 2M for 25 min at 37 °C and EdU detected following the manufacturer’s guidelines (Click-iT EdU, Life technologies). DAPI was used to counterstain nuclei. Pictures were acquired using an automated Zeiss ApoTome or confocal (LSM 780) microscopes (Carl Zeiss) and maximal intensity projections of three optical sections (10 μm thick in total) taken and quantified using Photoshop CS5 (Adobe). In all cases, GFP immunohistochemistry was performed to assess infectivity, which was confirmed in the vast majority (>90%) of the cases. Neurogenesis quantifications were performed on one complete DG series per animal considering the subgranular zone (NSC) or the whole thickness of the granular cell layer (immature and mature neurons) of both the dorsal and ventral hippocampus, whereas neuronal activity (c-Fos) quantifications were restricted to representative sub-areas of the dorsal hippocampus (−1.3 to −2.3 from Bregma) selected in the DAPI channel and identified anatomically (Allen Brain Atlas). Quantifications in the striatum were performed in the lateral and medial portions of the dorsal caudate/putamen (+1.1 to 0.0 from Bregma). Areas were measured using Fiji 1.45b (ImageJ) and morphometric analyses performed on confocal reconstructions through the entire section to include most processes that were later traced and visualised using Fiji (ImageJ).

### Electrophysiology

One twisted wire tetrode per CA1 hemisphere was implanted in mice anesthetized with a 50 mg/kg pentobarbital supplemented by 20% every hour. The bilateral craniotomies were performed using similar coordinates as described above and a 0.2 mm diameter drillbit (Edenta). Insertions were performed with a micromanipulator with a trajectory perpendicular to the dorsal skull surface until detection of pyramidal cells identified by their units characteristic firing patterns. UV curing glue was used to fix the tetrodes to the skull and cover the craniotomies. Three bone screws (0.5 mm) and dental cement were used to reinforce the implantation prior to closing the incision. After 48 h recovery, LFPs were recorded for 50 min during free exploration in silent and dimly lighted conditions (150 lux) using a RHD2000 Evaluation System and a RHD2132 Amplifier Board (INTAN Technologies) at a sampling rate of 30 KHz. Data were analysed off-line and ripple detection carried out by applying a FIR filter in the 110–240 Hz range^62^ converting the oscillation amplitudes to absolute values. In order to analyze only ripples occurring during a natural drowsy state, periods of overt sleep (featuring marked increase in theta-frequency activity) were detected and discarded, as well as the 10 s worth of recordings preceding and following them, to exclude any ambiguous states. No differences were observed in arousal between 4D and control mice. Next, a permissive detection threshold was initially set for the absolute amplitude traces and a ripple database generated and manually curated to eliminate false positive or negative events by three experimenters blind of the manipulation. We set an upper cutoff criterion of 15 s, such that outliers and long inter-ripple intervals from state changes did not excessively influence outcome. Tetrode location was confirmed post-mortem (Supplementary Fig. 2D). Analyses were performed considering >1500 ripples with duration being assessed on the number of full oscillation cycles based on the zero crossings of the FIR filtered trace when the absolute amplitudes were above detection threshold. Ripple internal frequencies were calculated as length divided by the number of full oscillations (Fig. S2C). A non-parametric Kruskal–Wallis test was used to assess significance among groups, due to the non-normal distribution of the data, and using Wolfram Mathematica 11.3 (Wolfram Alpha LLC) for analyses.

### Assessment of navigation

An elevated Barnes maze (1 m diameter) was used containing twenty holes (10 cm diameter) on the periphery one of which was connected to an escape box. Experiments were performed in a noise-isolated room with strong illumination (300 lux) and consisting of three phases: habituation (1 day), learning (4 days) and reversal (3 days) and adapting a paradigm already described for young mice in the Morris water maze^49,50^. Briefly, after acclimatisation to the testing room (>30 min) sessions were recorded using the Ethovision system (Noldus) and the platform and escape box cleaned with 70% ethanol at the end of each trial with mice completing the test being placed in an individual holding cage. On the habituation day (two trials) mice were placed in the centre of the maze and given 4 min to freely explore after which they were guided into the escape box and kept there for 30 s. During learning (three trials of 3 min with >30 min inter-trial interval) mice were trained to find a new position of the escape box. In the reversal phase (same as learning) the escape box was relocated at 180°. Probe tests consisted of a single trial for 2 min and without escape box. Navigational strategies were assessed by analysis of the searching trajectories and following the criteria previously described in testing young mice^49,50^ with the exception that searching at the periphery of the maze (previously defined thigmotaxis) was now considered chaining due to the different distance of the target to the centre of the maze. Every trial was scored according to the predominant strategy by two experimenters blind of treatment. Latency (time to the first encounter with the target) and pathlength (total distance travelled) were calculated using Ethovision’s software.

### Fear conditioning

Fear conditioning and contextual discrimination were assessed using a Multiconditioning System apparatus (TSE). Mice were acclimatised as described above and placed into a shuttle box as either context A (soundproof with two opposing transparent and two dark plexiglass walls illuminated from above and ventilated) or B (black–white striped plexiglass walls without ventilation and illuminated through doors left ajar). An electric foot-shock (2 s, 0.75 mA) was delivered only in context A through a steel grid after 3 min and the mouse returned to a holding cage 15 s later. Grids and shuttle box were cleaned either with 70% ethanol or non-alcoholic antiseptic solution prior to exposure to context A or B, respectively. A-B vs. B-A exposure orders were performed (Supplementary Fig. 4A) with a minimum interval time between trials of 2 h. Levels of freezing were measured using the manufacturer’s software and assessment of discrimination ratio between contexts calculated as freezing in (*A* − *B*)/(*A* + *B*) with a score of 0 or 1 representing a complete lack or perfect discrimination, respectively. Probe trials lasted 3 min without foot-shock. Re-training was performed in the same conditions as training.

### Statistics

Quantification of cell types and morphometric analyses were performed on independent biological replicates (see respective figure legends for *N* = mice and *n* = cells counted per mouse) and depicted as means ± SDs or box–whisker plots (always depicting the median, first and third quartile and inner fences) respectively, with significance being assessed by two-tailed, unpaired Student’s *t* test. Behavioural analyses were performed on 8–11 mice per group (as indicated in figure legends) and data depicted as area charts (navigational strategies), mean ± SEM (latency, pathlength, discrimination ratio) or box–whisker plots (contextual discrimination, perseverance and probe tests). Statistical significance was accordingly calculated by Wald-test of odds ratios assessed by logistic regression (navigational strategies), two-way ANOVA (evolution of performance over trials/days) or two-tailed paired or unpaired (as appropriate) Student’s *t* test (difference in performance in specific trials/days). Sharp-wave ripples were analysed by the non-parametric Kruskal–Wallis test as described above.

### Reporting summary

Further information on research design is available in the Nature Research Reporting Summary linked to this article.

## Supplementary information

Supplementary Information

Peer Review

Reporting Summary

## Data Availability

All relevant data are available from the authors upon request.
