# Author Correction: Increasing neurogenesis refines hippocampal activity rejuvenating navigational learning strategies and contextual memory throughout life

**DOI:** 10.1038/s41467-020-14935-4

**Published:** 2020-02-25

**Authors:** Gabriel Berdugo-Vega, Gonzalo Arias-Gil, Adrian López-Fernández, Benedetta Artegiani, Joanna M. Wasielewska, Chi-Chieh Lee, Michael T. Lippert, Gerd Kempermann, Kentaroh Takagaki, Federico Calegari

**Affiliations:** 1 CRTD—Center for Regenerative Therapies TU Dresden, Technische Universität Dresden, Fetscherstrasse 105, 01307 Dresden, Germany; 2 Leibniz Institute for Neurobiology, Magdeburg, Brenneckestr. 6, 39118 Magdeburg, Germany; 3 German Center for Neurodegenerative Diseases (DZNE), Tatzberg 41, 01307 Dresden, Germany; 4 Laboratory in Sensory Physiology, Institute of Biology, Otto-von-Guericke University Magdeburg, Leipziger Strasse 44, 39120 Magdeburg, Germany; 5 Present Address: Hubrecht Institute, Developmental Biology and Stem Cell Research, Uppsalalaan 8, 3584 CTUtrecht, The Netherlands

**Keywords:** Cognitive ageing, Hippocampus, Adult neurogenesis, Neural stem cells

Correction to *Nature Communications* 10.1038/s41467-019-14026-z, published online 09 January 2020.

The original version of this Article contained errors in the author affiliations. Affiliation 2 incorrectly read Laboratory of Integrative Neuroscience, Institute of Biology, Otto-von-Guericke University Magdeburg, Leipziger Strasse 44, 39120 Magdeburg, Germany. This has been corrected to Leibniz Institute for Neurobiology, Magdeburg, Brenneckestr. 6, 39118 Magdeburg, Germany. Also, the affiliation of Kentaroh Takagaki with Laboratory in Sensory Physiology, Institute of Biology, Otto-von-Guericke University Magdeburg, Leipziger Strasse 44, 39120, Magdeburg, Germany was inadvertently omitted.

These have now been corrected in both the PDF and HTML versions of the Article.

