# Supplementary material for: Increasing neurogenesis refines hippocampal activity rejuvenating navigational learning strategies and contextual memory throughout life
Source: Nat Commun. 2020 Jan 9;11:135. doi: 10.1038/s41467-019-14026-z (PMC6952376; doi:10.1038/s41467-019-14026-z)
Supplement: Supplementary file 1 — Supplementary Information [file 41467_2019_14026_MOESM1_ESM.pdf]

Supplementary Information

Increasing Neurogenesis Refines Hippocampal Activity Rejuvenating Navigational Learning Strategies and Contextual Memory Throughout Life

Berdugo-Vega et al.

Supplementary Fig. 1: 4D increases the generation of morphologically normal neurons

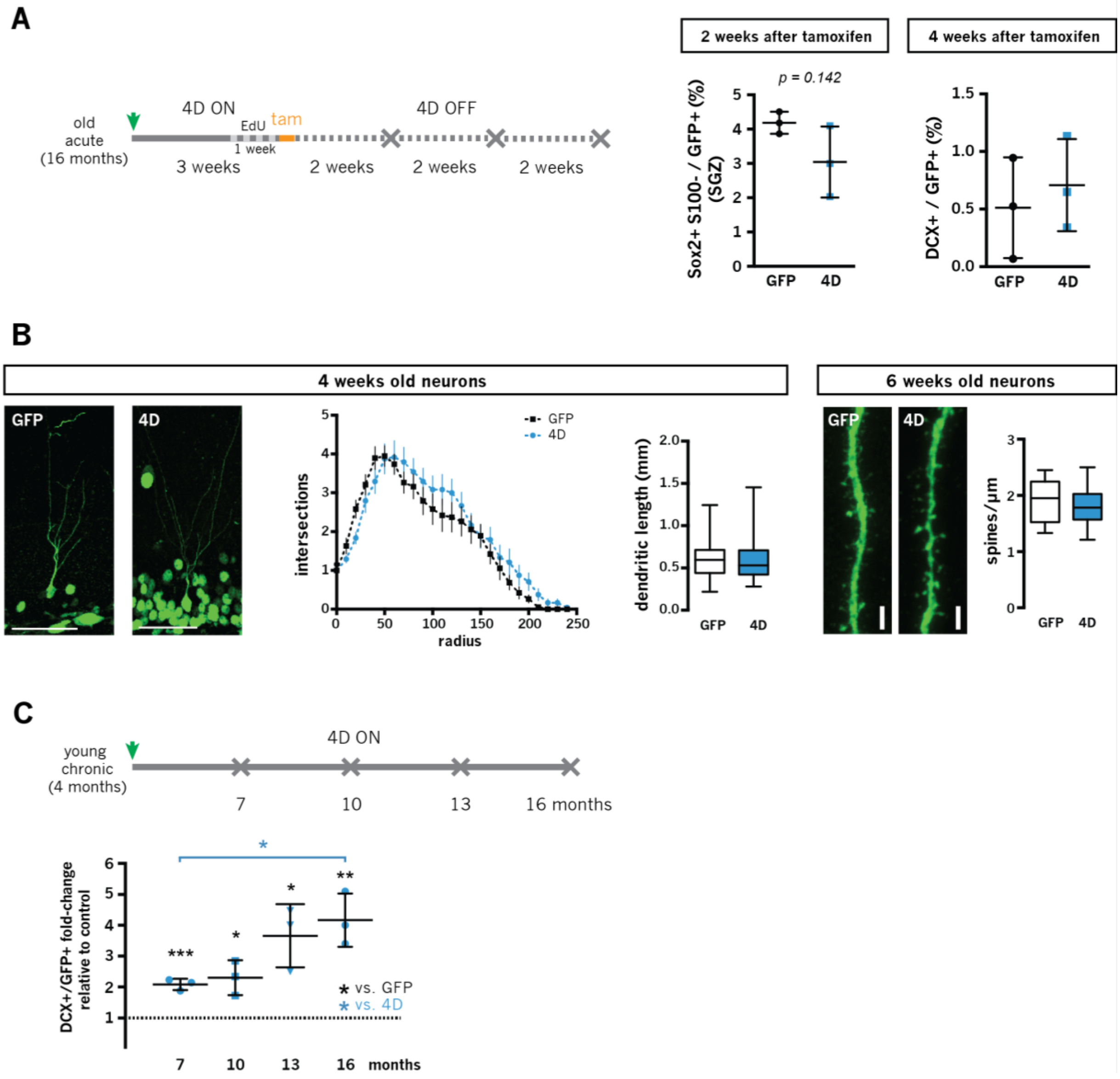

Supplementary Figure 1.

4D increases the generation of morphologically normal neurons. A) Experimental layout (left) and cellular quantifications during acute 4D manipulation to address NSC and neurogenesis normalization at 2 and 4 weeks after tamoxifen, respectively. **B)** Immunohistochemistry fluorescence pictures and morphometric analyses to assess integration and maturation of 4D-derived newborn neurons 4 (left) or 6 (right) weeks upon tamoxifen administration (as depicted). Note the equivalent Sholl profiles (means±SEM), dendritic lengths and spine densities (box-whisker plots) of GFP (white) and 4D (blue) derived neurons consistent with a previous report from our group [30] and showing that 4D-derived neurons can mature and integrate as physiologically generated adult-born neurons. **C)** Quantification of the fold-increase in neurogenesis relative to controls (as indicated; data correspond to that in Fig. 1D). Note the progressing increase in the magnitude of the effect of chronic 4D overexpression with time, whose significance is expressed as relative to control (black) or 4D (blue). N=3; n>1000 (A), >18 (B, left) and >1500 (B, right); * p<0.05, ** p<0.01, *** p<0.001 assessed by unpaired Student's t-test. Scale bars=50 µm (B, left) and 2 µm (B, right).

Supplementary Fig. 2: Effects of neurogenesis in the trisynaptic hippocampal circuit

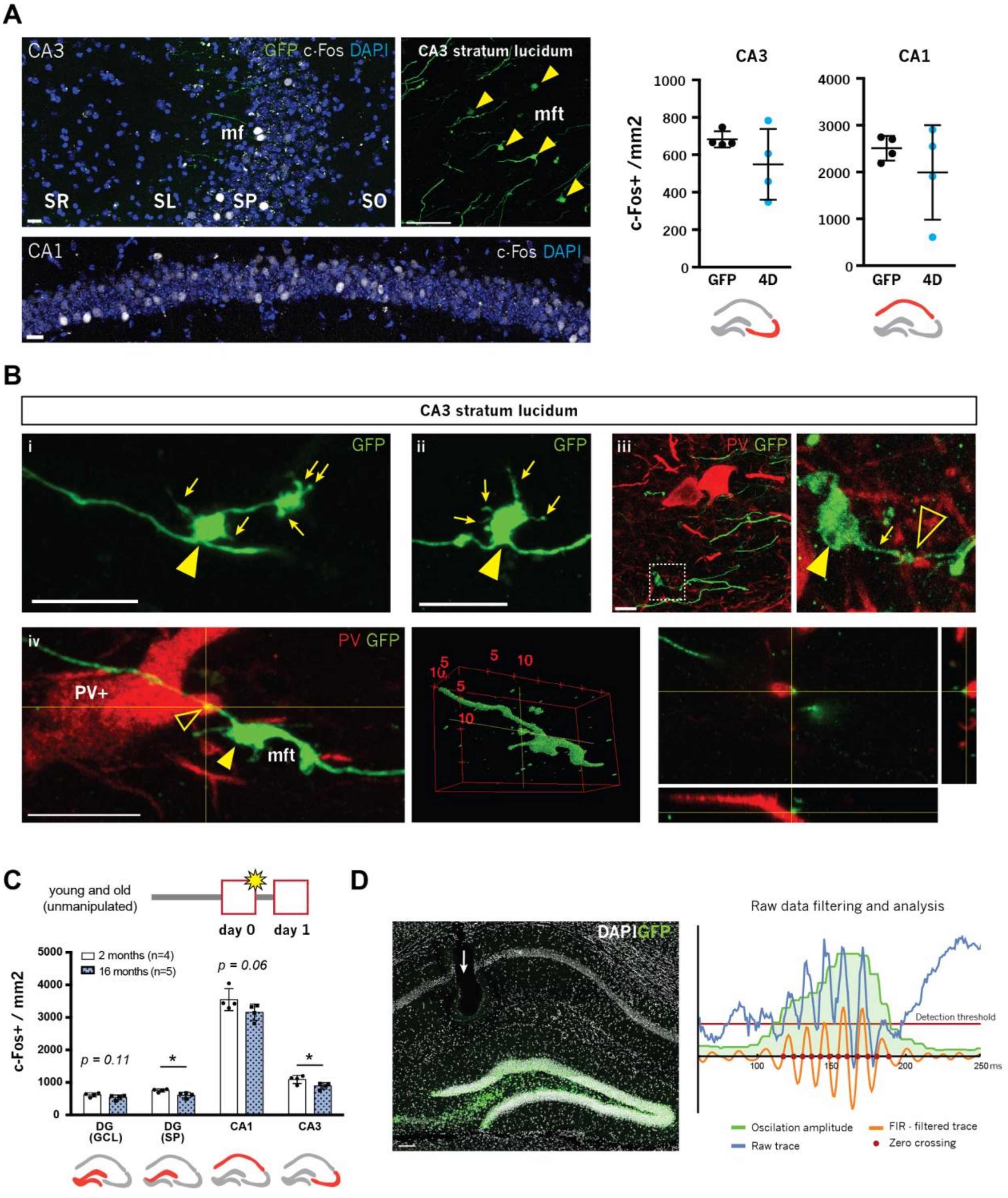

Supplementary Figure 2.

Effects of neurogenesis on the hippocampal trisynaptic circuit. A) Immunohistochemistry fluorescence pictures (left) and quantifications (right) for c-Fos+ cells in CA3 and CA1 (as indicated). Note the presence of GFP-cytoplasmic mossy fibers (mf) and terminals (mft, filled arrowhead) in the stratum lucidum of CA3. Data were expressed as c-Fos+ cells per area scored in the respective structure of the dorsal hippocampus. N=4. **B)** Immunohistochemistry fluorescence pictures depicting the morphology of GFP+ mossy fibers terminals and highlighting the presence of emanating filopodia (yellow arrows, top) contacting PV+ processes (empty arrowheads, right and bottom). Specifically, pictures in the bottom represent the maximal intensity projection, 3D-reconstruction and single confocal plane containing a filopodial-PV contact (left to right, respectively). **C)** Experimental layout and quantifications of memory-induced expression of c-Fos showing a general reduction in activity in different hippocampal areas of old (16 months, grey) relative to young (2 months, white) unmanipulated mice. **D)** Fluorescence picture of the mouse hippocampus upon immunohistochemistry for GFP (green) and DAPI counterstaining (white) to assess the correct implantation of a tetrode in the pyramidal layer of the CA1 (white arrow). The same procedure was used for all mice used for generating data shown in Fig. 2E. On the right, drawing illustrating the analysis of an LFP trace (blue) containing a ripple. The points at which the FIR-filtered trace (orange) crossed the X axis, or zero crossings (red dots), were used to calculate the number of full oscillations for any given ripple whose length was defined as the time elapsed between the first and last oscillation with amplitudes above detection threshold (green and red lines, respectively). Ripple internal frequencies were calculated as ripple length divided by the number of full oscillations. Scale bars=20 (A), 10 (B) and 100 (C) μm. * $p<0.05$ assessed by unpaired Student's t-test (C).

Supplementary Fig. 3: Establishment of the Barnes maze to test navigational strategies

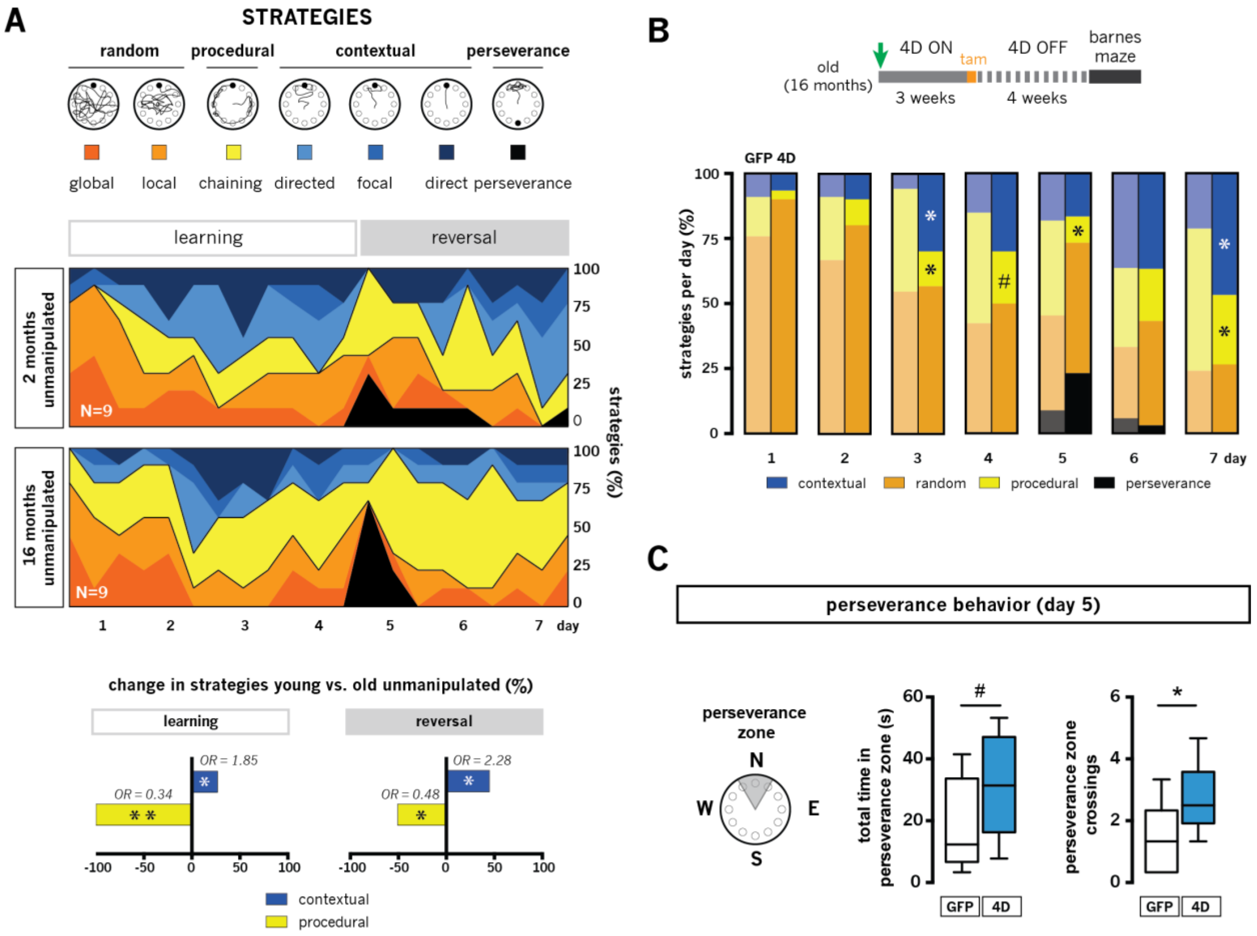

Supplementary Figure 3.

Establishment of the Barnes maze to test navigational strategies. A) Color-coded searching tracks (top) and their relative contribution and significant change (middle and bottom, respectively) during 7 days of learning and re-learning of unmanipulated young and old mice (as indicated) and validating the use of our approach to assess navigational strategies upon increased neurogenesis (Fig.3B and 4E). **B)** Navigational strategies of 4D and GFP-treated mice shown in Fig. 3B generated by depicting the contribution of grouped strategies (as indicated) each individual day of testing. **C)** Box-whisker plots depicting the perseverance behavior (black in B) of mice tested as in Fig. 3B and 3C supporting the acquisition of a place memory in 4D-treated mice. N=9 (A); N=11 and 10 for GFP and 4D respectively (B and C). # $p<0.1$, * $p<0.05$, ** $p<0.01$ assessed by Wald test (A and B) and unpaired Student's t-test (C).

Supplementary Fig. 4: Establishment of fear-conditioning paradigm to assess discriminatory vs. procedural learning

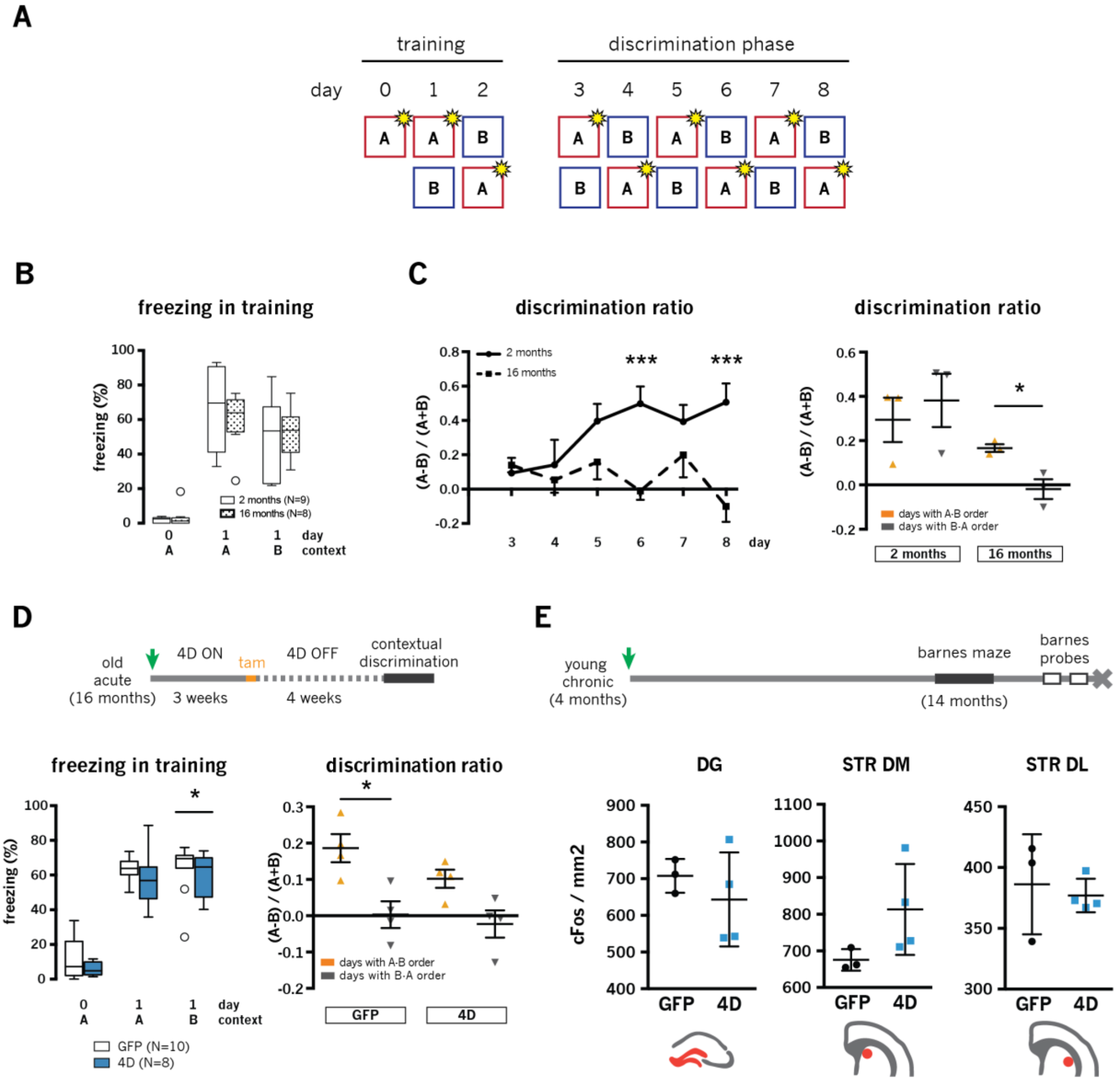

Supplementary Figure 4.

Establishment of a fear-conditioning paradigm to assess discriminatory vs. procedural learning. **A)** Schematic representation of a fear-conditioning test consisting of a training and discrimination phase in which presentation order of 2 contexts was alternated each day. Foot-shocks are depicted (yellow). **B-C)** Box-whisker plots (B) and discrimination ratios (C) representing the freezing response of 2 and 16 months old, unmanipulated mice during training (B) or testing (C). Note the progressing discriminatory performance of young but not old mice (2-way ANOVA: time: $F_{(7,105)}=1.23$, $p=0.293$, group: $F_{(1,15)}=48.35$, $p<0.0001$, interaction: $F_{(7,105)}=2.69$, $p=0.013$; C, left) and, in particular, the differential effect of presentation order with old mice only showing positive discrimination in A-B but not B-A days (C, right). **D)** Experimental layout (top), box-whisker plot quantification during training (bottom, left) and discrimination ratio during discrimination phase (bottom, right) used to assess contextual learning upon acute increase in neurogenesis in 18 months old mice by the test depicted in A and in which 4D-treated mice showed a reduced generalization (bottom, left) and bias for presentation order (bottom, right). **E)** Experimental layout (top) and quantifications (bottom) for total c-Fos+ cells per mm^2 assessed in the DG, dorsomedial and dorsolateral striatum (STR DM and DL respectively; representative pictures can be found in Fig. 4G) of GFP and 4D chronically-treated mice and probe tested for memory 6 weeks after the Barnes Maze. N=8 and 9 (B and C); n=3 (C, right, representing days with A-B or B-A order); N=8 and 10 (D); n=4 (D, right); N=3 and 4 (E). * $p<0.05$, *** $p<0.001$ assessed as paired or unpaired Student's t-test (for intra or inter-group comparisons respectively).

Supplementary Table 1. List of primary antibodies

antigen	dilution	manufacturer	catalogue #
BrdU	1:250	Abcam	ab6326
c-Fos	1:1000	Synaptic Systems	226003
Dcx	1:100	Santa Cruz Biotechnology	Sc-8066
GFP	1:500	Thermo Fisher	A-11122
NeuN (Fox3)	1:500	Abcam	Ab104225
Pv	1:5000	Swant	pv235
S100β	1:1000	Abcam	ab14688
Sox2	1:500	Santa Cruz Biotechnology	Sc-17320

List of antibodies Form left to right: antigen recognized, dilution used, provider and catalogue number of primary antibodies. In most cases, Alexa Fluor secondary antibodies (Jackson ImmunoResearch) diluted 1:1000 were used.
