# Supplementary material for: Increasing neurogenesis refines hippocampal activity rejuvenating navigational learning strategies and contextual memory throughout life
Source: Nat Commun. 2020 Jan 9;11:135. doi: 10.1038/s41467-019-14026-z (PMC6952376; doi:10.1038/s41467-019-14026-z)
Supplement: Supplementary file 2 — Peer Review [file 41467_2019_14026_MOESM2_ESM.pdf]

Editorial Note: Parts of this Peer Review File have been redacted as indicated to maintain the confidentiality of unpublished data.

Reviewers' comments:

Reviewer #1 (Remarks to the Author):

Kempermann

General Comments

This manuscript describes the use of a tamoxifen-controlled viral-induced increase in neurogenesis, and the effects of such an increase on a number of parameters of brain function ranging from cfos activation through to behavior. The idea of doing this ingenious, but the manuscript suffers from a lack of clarity throughout, starting with the abstract, and more seriously in the results.

I do not know if this is rescuable or not, but my fear is that the claims of Figure 2 on sharp waves are simply not credible (Points 8 and 9 below). If so, it would be essential for additional experiments to be conducted to ensure the result is sound and shown more clearly than at present. Given this, the Discussion at the end is not credible.

1. Abstract – is very abstract. Too little information is given about what was actually done or found with only theoretical interpretations made about the undeclared results.

2. Page 2 "…but their neurobiological mechanisms are hardly understood." This is an unreasonably strong statement given the wealth of work done on the physiology of ageing, especially in the dentate gyrus, by Carol Barnes, Michela Gallagher and others (of which the former is cited but with apparently little knowledge of what she has actually shown) A great deal is known about neural compensation for the loss of afferent input to the dentate gyrus with ageing and to imply that we know next to nothing is seriously impolite.

3. Page 4 "…rescue the switch from contextual to procedural learning". This is a negative way to put it. Why not "…promote the use of more effective contextual learning and spatial navigation strategies"?

4. Page 4 "Cdk4/cyclinD1 increases NSC expansion and neurogenesis throughout life." I think explicit mention of this approach should be made in the Introduction and not merely mentioned for the first time in the Results. The approach should also be explained as few readers will have time to pause to go back to the earlier reference using this technique at this stage of reading a paper.

5. Page 4, para 2 of results. If I understand things correctly, this viral construct causes a transient 6-fold increase in cell proliferation and a 2-fold increase in neural stem cells, leading to measureably higher numbers of immature neurons. However, I do not have a sense of how these numbers tally with the existing number of adult neurons – is it 0.1%, 1%, 10% - it is very unclear. The radical increase of something that is too rare to matter much is very different from a substantial increase in the number of immature neurons that are used one month later once their connectivity is established.

6. Page 4 bottom. "Acute 4D overexpression (together with GFPnls) in 16 months old mice for 3 weeks resulted in a 6-fold increase in overall proliferation and active NSC among targeted (GFP+) cells relative to controls infected with GFPnls viruses (EdU+: 0.77±0.22 vs. 0.16±0.11% and EdU+Sox2+: 0.53±0.14 vs. 0.08±0.07%; $p=0.014$ and $p=0.008$, respectively), which was paralleled by a doubling in NSC (Sox2+S100β−: 6.17±1.1 vs. 3.73±0.74%; $p=0.033$) (Fig. 1A)." Neither the figure legend nor the text is very clear in taking the less initiated reader through what is being shown here. The reference is to Figure 1A. The text says "increase in overall proliferation" – this is not explicitly referred to in the Figure. The text says things are bigger in GFP+ cells relative to controls with GFPnls viruses. The figure has different colour symbols for GFP and 4D groups, with GFP being in fact the control group The text says "paralleled by a doubling in NSC". But, again, neural stem cells are not referred to explicitly in the figure. So – while I do not doubt the assertions – the text is one thing and the figures to which the text refers are another, with one or both being written in "lab jargon" that is not explained to the reader. Finally, there is a classic example of looking at the data from two different premises in Figure 1D. The text states that the persistent neurogenesis is 4-fold higher in the older animals who have had a longer time in the chronic study ("… However, the absolute value of the ratio

of DCX+ to GFP+ declines with age to a value of < 1% in the older animals. Thus, a reader is required to look at the declining absolute level of the ratio in Figure 1D to derive mentally that the relative ratio is increasing. This is OK (I guess) but hellishly confusing.

7. Am I right in worrying that there is a confound between acute vs chronic treatment, and old vs young? This may not matter, but it is either unfortunate or the reasons for doing this not well explained. Perhaps it's fine.

8. Page 8: "..and supports the notion that neurogenesis may play a role in memory consolidation (32)." The notion that neurogenesis plays a role in memory consolidation seems extremely unlikely, if only for timing reasons. If neurogenesis is triggered by a learning episode, the proliferation, integration and connectivity of the new neurons takes about a month. This is too long a time before the rate at which memory consolidation could be changed, as much of it would already be done, and thus hard to see how it could possibly have much impact. I guess greater sparsity of representations and more immature dentate neurons might affect the efficiency of memory consolidation guided by CA1 sharp waves, but this could not be in any way relevant to the information that actually triggered the neurogenesis. This idea is surely a non-starter except as a non-specific effect.

9. In this context, the data of Figure 2D are simply not credible. It is claimed, in both the title of the figure and the detailed text, that neurogenesis triggers a decrease in activity. A decrease is seen in cfos, but the electrophysiology shows no such effect with massive variability and overlap between the control and experimental conditions. The assertion that these data are significant at the p = 1 x 10 to the 10 level (eg. Fig 2D right) cannot be an accurate description of the data that is shown. Note also that the n numbers are simply too small for comfort with group sizes of N=3 to N=6. This is of major concern.

10. Page 10 "Consistent with the known effect of aging on navigation (7-9), young mice displayed a greater use of contextual (allocentric) relative to procedural (egocentric) strategies than old mice throughout both the learning and reversal phases (odds ratio young vs. old: contextual=1.85 and 2.28, procedural=0.34 and 0.48; Wald-test p=0.033 and 0.002; respectively; Fig. S3B and S3C)." Given the importance of the claim, the deposition of these poorly explained findings in the supplementary feels wrong. A further difficulty is the failure to explain with any clarity how the difference between navigational strategies is identified, measured or tested. A figure of what are claimed to be different strategies is shown in Figure 3 but it is unclear how actual paths are analysed with respect to these exemplars. Is a single path classified as one or another? Or might a strategy change half-way through a trial? The data analysis is very vague. To add that the data appear to show that the mice appear to be moving at between 4 and 6 cm/sec. This feels slow to me for mice in a typical open-field unless there is a lot of stopping – but again maybe it is OK.

11. I am not making on comments on Figure 4 because, not fully understanding Figure 3, I do not feel able to comment on Figure 4.

12. Discussion: I find it difficult to follow the strand of the Discussion, with claims that (a) strategy choice rather than the efficacy of any one strategy may be what matters – though the data behind this claim are largely descriptive, and (b) that increasing NSC results in a change in sharp wave ripples which I have previously noted seems not to be shown in the results of Figure 2.

Reviewer #2 (Remarks to the Author):

Berdugo-Vega and colleagues present an interesting set of findings exploring the contributions of enhanced and inducible adult neural stem cell (NSC) expression in the hippocampus in adult mice to behavior and physiology. Specifically, this study implemented virally-mediated Cdk4/cyclinD1 (4D) overexpression (Please use the term retrovirus-this is absent from the entire manuscript) to significantly enhance NSC expression in aged animals, but also in younger animals (e.g., in 7 months

of age). 4D overexpression in the hippocampus resulted in changes in physiological readouts of the hippocampus function (e.g., alterations in CA1 LFP signals and behavior-induced c-fos expression in the dorsal hippocampus) while also altering behavioral strategies in aged mice seen in various navigational tasks. Aged mice normally become more dependent on procedural strategies (which may be a detriment to context-dependent behavior and result from loss of normal hippocampal function), but aged mice treated with 4D appeared to rely more heavily on contextual cues after learning, suggesting that it may rescue some hippocampal function via facilitation of NSC induction. Moreover, improvements in context-dependent behaviors were observed even if chronic 4D treatment was applied in young mice that were later tested after aging (such effects may relate to interaction with activity in the striatum, at least based on c-fos analyses). Overall, this is an interesting study that attempts to link increases in adult hippocampal neurogenesis with circuit properties and behavior. However, the authors try to cover too much ground leaving out key comparisons and controls and making the interpretation of the data very difficult.

Primary concerns:

What are the levels of NSCs and progenitors in these aged Nestin mice prior to 4D injections?

The 4D Off design is hard to interpret with the information provided. This is because the 4D ON cells differentiate into adultborn neurons even in the absence of TAM (Many of the 4D expressing GFP cells appear to be in the GCL at 3-4 weeks post infection (Fig.1A) and so the net population of extra neurons (generated after TAM) are of many different ages. So the 8 week timepoint is likely to have neurons that are less than 8 weeks of age. Please discuss adequately.

I am not convinced that the 4D OFF system reverts levels of neurogenesis to baseline. Because of small overlap between Nestin CreeRT2 recombined cells and activated stem cells and progenitors that are infected by the 4D retrovirus (at 3 or 4 weeks post infection of 4D virus), the frequency of CrERT2 deletion of NLS and 4D cassette is likely to be very low. This means that escaper 4D ON cells will continue to generate new neurons and will be very hard to detect since aged mice show extremely low levels of stem cell activation. Relatedly, do neural stem numbers return to baseline in 4D OFF experiments? Please show Nestin RGL GFP following TAM in 4D OFF condition.

Please provide data for 4 and 6 week YFP neurons rather than just DCX and Edu/NeuN.

Provide total DCX counts for Fig. 1B analysis. Total counts give a sense of how neurogenesis is changing in the hippocampus. If recombination efficiency is very low, then it is likely that there are very few newly added neurons. This is important information for the reader. The authors do discuss the very low numbers (approx. 100 extra DCX cells) in aged mice but this data (exact numbers) needs to be plotted and conveyed to reader.

Fig. 1C: Brdu/NeuN/GFP overlap: The NeuN signal within dotted line is not convincing.

Fig. 1D. What is the effect of chronic overexpression for 3, 6, 9 months on size of NSC pool and total count of 4 week old neurons at each of these timepoints selected for behavior?

I appreciate that the authors are trying to compare DG c-fos levels relative to CA3 and CA1 (as an indicator of relative excitation in the network) in Figure 2B, however, the mean values of CA3 and CA1 should also be reported. For example, although c-fos levels (per mm2) in DG appeared lower, that doesn’t exclude the possibility that CA3 or CA1 c-fos levels are unchanged (relative to controls); moreover, the lower values of DG/CA1 divisions shown in Fig. 2B could actually be biased by higher levels of CA1 (or CA3) relative to controls, thereby generating lower values for DG/CA3 or DG/CA1 calculations. Similar claims can be made about the c-fos data shown in Figure 4G; reporting DG, DM, and DL values (in addition to the division analyses) will greatly aid in the interpretation of the relative impact of 4D induction on the network.

Fig.2B. Essential comparisons (Young mice and learning naïve mice versus aged mice) are missing to support authors’ claims. The authors must provide cfos data for aged and young learning- naïve mice. Also, it is simply not clear from the data that the cfos changes reflect “sparsity of memory representations”. This claim needs to be addressed using memory tagging approaches or in vivo

imaging of memory ensembles. This phrase should be should be removed from manuscript.

I believe the authors need to clarify further what is meant by testing for electrophysiological recordings in animals during a "natural drowsy state" (equivalent time of day for the mice?, etc.). I appreciate that testing was done in a dimly lit conditions, but it is difficult for the reader to determine if differences in inter-ripple intervals (for example) can be attributed to the 4D treatment, or if there were potential differences in the levels of arousal at test that account for the findings in 4D vs. controls (i.e., perhaps the inter-ripple intervals were shorter in controls because they exhibited more of them during the session). Can the authors indicate whether 4D vs. controls differed in the total number of identified ripples for the recording sessions?

Previous work has shown that amplitude and frequency of SWRs increase in CA1 after contextual fear conditioning. How does the authors manipulation affect SWRs following learning?

The authors observe a dissociation between behavioral measures and search strategies. This is interesting but requires greater discussion. For example, Fig. S3E and Fig. 3C argues against improved reversal learning as 4D mice show increased perseverance behavior. According to the latency plots, the controls do not exhibit increased latency when reversal begins. Previous studies have reported a congruence between search strategy and behavior. Please discuss further.

Fig. S4E-F: n=4-5 mice per group is not sufficient for meaningful interpretation of behavior especially, when there is also variability related to viral injections and TAM mediated deletion etc…

Do the authors see an effect of 4D expansion on forgetting?

Other concerns:

1) Figure 2A reports n's of 5-6 for freezing behavior (which are noted as the basis for Figure 2B), but the graphs in Figure 2B only show 4 animals per group. Can the authors clarify/fix?
2) In the main text and figures, the authors list significant p values, but without showing the exact F (or t) values for these tests or their degrees of freedom (for example) that led to that p value. I recognize the methods state that tests were used as appropriate, but without showing such information, the reader must infer any main effects or interactions, and thus cannot tell if post-hoc comparisons are used appropriately.
3) 4D induction depends on successful incorporation of the virus in the DG. There is no mention of whether animals were or were not excluded from experiments based on successful "hits" or "misses" of viral injections. I assume the authors have done their due diligence in this regard, but it should be stated if and how the authors identified successful 4D or GFP infection in their experiments.
4) c-fos is stated as counts from granule cell layer of DG; is this from the dorsal, ventral, or both blades? Noting such locations may also be important because representative images in Figure 1 (for the DCX quantifications) appear to show cells from the medial DG (with both blades) but other images just show a single blade, so it's not clear where quantifications are being made or if we should have any expectations of differences in these counts at various medial-lateral segments of the DG. Also, if possible, and to better understand how representative these counts may be, it may benefit readers to know the size (expressed as mm2, for example) of the domain quantified for each subregion.
5) In Figure 1B and 1D, is there a reason we are only shown DCX+/DAPI+ in the images, given that the authors are showing data comparisons for DCX+/GFP+ percentages? Also, the yellow tick marks are very small, and not easily discernable unless the reader zooms in quite a bit. I doubt it will show up well in a printed version, I would recommend making the images larger, and/or making yellow arrows larger.
6) I will leave this up to the discretion of the authors, but it's unclear to me why box-and-whisker plots are used for some data presentations while mean/SEM (though sometimes they are stated as SD) (with individual data points) are shown for others. Certainly, the some of the behavioral data could be represented with means/SD/SEM, thus allowing reader to see individual performance in these tasks via individual data points.
7) The use of "old people" in the text may be construed as offensive; I recommend changing.

Reviewer #3 (Remarks to the Author):

Major points

- The authors claim that the expansion of neurogenesis counteracts the effects of aging on several aspects related to hippocampal functioning. However, only old animals have been used in most of the analyses shown. Thus, it is not possible to conclude that this manipulation counteracts the effects of aging unless the effects were tested on young mice too. In this regard, cfos+ cell counts, electrophysiological recordings, and behavioral test should be performed on both young and aged mice (GFP and 4D groups), in order to correctly interpret the effects of 4D overexpression on aging. Otherwise, no conclusions regarding the effects of 4D overexpression on the changes triggered by aging can be reached. If authors do not wish to consider performing these experiments, the whole manuscript should be re-focused. E.g., referring to rejuvenation navigating strategies would not be possible, given that the control effects of 4D on young animals is unknown.
- The expression of cfos should be analyzed in naïve animals not subjected to behavioral tests. Moreover, absolute numbers of cfos+ cells for every anatomical region should be provided in all the cases.
- This Reviewer believes that there are several inconsistencies regarding the statistical analyses performed. For example, was normality determined throughout the manuscript? Which statistical test was used? Were all the variables tested normal? Why was SD represented in cell counts whereas SD was used to represent behavioral analyses? Moreover, repeated measures ANOVA tests should be used for behavioral analyses that are performed on the same mouse over consecutive days.

Minor points

ABSTRACT

- In the last sentence of the Abstract, the authors affirm that the results were obtained "by exploiting the brain's endogenous reserve of neural stem cells". However, this Reviewer believes that, given that a transgenic mouse + viral injections have been used, referring to the endogenous reserve of stem cells is not totally appropriate in this context.

INTRODUCTION

- In addition to feedfodward inhibition to mature neurons, the authors may wish to cite other aspects of newborn neuron behavior that are important for hippocampal functioning.
- In page 3, authors affirm that "In contrast to behavioral performance, effects of neurogenesis on learning strategy choice were never explored". They migh wish to cite Garthe et al. 2009, PMID 19421325.
- The concept of hippocampal-estriatal memory competition has not being correctly introduced. Please expand its description and relevance for the present work.
- Also, the mouse model that is going to be used should be described in the Introduction section.

MATERIAL AND METHODS

- In Fig S1, the number of cells counted per mouse should be indicated in the Figure legend.
- The type of viruses (lenti, AAV, …) that have been used should be specified.
- Methods to perform cell counts should be described into much more detail.

RESULTS

- The images shown in Figure 1 D are of poor quality. Please provide better images at low and high magnification showing the increase in the number of DCX+ cells.
- In Fig 2, the total numbers of cfos+ cells for the CA1 and CA3 fields should be provided. In Figure 2, all the graphs should be adapted to the same format.
- In S3 E, are these data obtained from old or young mice?.
- In Fig 4 G, images are of poor quality. It seems that almost all the neurons of the hemi-brain are positive for cfos. Authors should provide more convincing images of the cells that are being quantified.

Moreover, the nomenclature used to define striatal areas is not very standard. Are they analyzing the caudate/putamen/GP structures? If so, how were these regions anatomically identified?
- Examples of cfos+ cells are provided both in Fig 2 and 4. However, immunofluorescence or immunohistochemistry images are shown in Figure 4 and 2 respectively. Were different methods used to quantify these number of cells in different experiments? Do the authors consider that this could affect the number of cells detected? As previously mentioned, absolute number of cells should be provided instead of cell ratios.

DISCUSSION AND INTERPRETATION OF THE DATA

- In page 19, the affirmation that the increase in neurogenesis was performed "without systemic effects or interfering with the physiology of the niche or the neurons themselves" seems to be too heavy. These factors have not being analyzed in such depth in the present manuscript.
- In the same page, authors affirm that "key downstream effects on circuitry and balance with the striatal navigational memory system" have been identified. This affirmation is not correct, given that the hierarchy between these circuits has not been determined.
- The striatum is a complex structure, which participates in several circuits involved in memory. This Reviewer consider that the examination of cfos+ cells in two areas of this structure (which, as indicated in the Major points, should be much more precisely described) is not sufficient for reaching this conclusion.
- Please consider that stereotaxic surgeries are being performed when affirming that "our study is the first to alter this competition without surgical or pharmacological manipulations".
- The final sentence of the Discussion seems to be out of place.

REVISION BERDUGO ET AL., NCOMMS-19-16743-T

Authors' introductory statement:

We thank the reviewers for their thorough assessment of our work and many constructive comments. We are pleased that they found our study *"ingenious, novel and interesting"* and appreciate their criticism about a general lack of clarity in describing our study. As the reviewers made it clear, the complexity of our study requires a more thorough description that we attempted in this revised and significantly extended version. Together with this, the reviewers will find below a point-by-point reply to their comments and the addition of several novel experiments that we hope will be to their satisfaction. All relevant changes to our original version are highlighted in red text in the revised manuscript.

REVIEWER #1 (REMARKS TO THE AUTHOR):

This manuscript describes the use of a tamoxifen-controlled viral-induced increase in neurogenesis, and the effects of such an increase on a number of parameters of brain function ranging from cfos activation through to behavior. The idea of doing this ingenious, but the manuscript suffers from a lack of clarity throughout, starting with the abstract, and more seriously in the results.

I do not know if this is rescuable or not, but my fear is that the claims of Figure 2 on sharp waves are simply not credible (Points 8 and 9 below). If so, it would be essential for additional experiments to be conducted to ensure the result is sound and shown more clearly than at present. Given this, the Discussion at the end is not credible.

We thank the reviewer for the compliment of defining our work ingenious and agree on its lack of clarity. We now extended the whole manuscript considerably. Concerning Fig. 2, the reviewer states that s/he is *"afraid that that the claims on sharp waves are simply not credible".* We do appreciate and agree that the variance in these measurements are big, but this was expected and also reported by previous studies. The reviewer should also consider that these data, depicted as box-whisker plots, do not follow a normal distribution and therefore were analysed by non-parametric tests based on thousands of recorded ripples (see specifically our answer to point 9).

We have now re-analysed our data and added a new series of c-Fos quantifications in several relevant areas that support the finding of a reduced hippocampal activity and sharp-wave ripples. Notably, this included an increase in DG-CA3 feed-forward inhibition showing the direct contact between mossy fibres belonging to newborn neurons and a higher percentage of activated parvalbumin inhibitory interneurons in the CA3 in 4D mice (new Fig. 2C-D and S2A-B). All this supported our main conclusion about 4D reducing the excitability of the hippocampus. We refer the reviewer to the specific answer below and our revised manuscript addressing these aspects.

1. Abstract – is very abstract. Too little information is given about what was actually done or found with only theoretical interpretations made about the undeclared results.

The abstract was revised and more information added within the Journal's limit of 150 words.

2. Page 2 "…but their neurobiological mechanisms are hardly understood." This is an unreasonably strong statement given the wealth of work done on the physiology of ageing, especially in the dentate gyrus, by Carol Barnes, Michela Gallagher and others (of which the former is cited but with apparently little knowledge of what she has actually shown) A great deal is known about neural compensation for the loss of afferent input to the dentate gyrus with ageing and to imply that we know next to nothing is seriously impolite.

We regret our failure to fairly cover the literature and hope to have addressed this with an expanded introduction, discussion and nearly 20 new citations including toning down strong claims like the one pointed out by the reviewer.

3. Page 4 "…rescue the switch from contextual to procedural learning". This is a negative way to put it. Why not "…promote the use of more effective contextual learning and spatial navigation strategies"?

We have turned this into a positive statement as suggested.

4. Page 4 "Cdk4/cyclinD1 increases NSC expansion and neurogenesis throughout life." I think explicit mention of this approach should be made in the Introduction and not merely mentioned for the first time in the Results. The approach should also be explained as few readers will have time to pause to go back to the earlier reference using this technique at this stage of reading a paper.

Again, we apologize for the lack of clarity and have considerably expanded the description of our 4D system, its previous uses and including a new Fig. 1A depicting the approach.

5. Page 4, para 2 of results. If I understand things correctly, this viral construct causes a transient 6-fold increase in cell proliferation and a 2-fold increase in neural stem cells, leading to measureably higher numbers of immature neurons. However, I do not have a sense of how these numbers tally with the existing number of adult neurons – is it 0.1%, 1%, 10% - it is very unclear. The radical increase of something that is too rare to matter much is very different from a substantial increase in the number of immature neurons that are used one month later once their connectivity is established.

The reviewer raises a number of important points. To start with, concerning the difference in *"6 vs. 2-fold increase"*, one should bear in mind that *"proliferative activity"* and *"cell numbers"* must not necessarily correlate or change by a similar magnitude. For example, NSC may undergo faster cell cycles but their number remain the same, or even decrease, depending on whether or not they divide to generate neurons or additional NSC. Not least, *"proliferative activity"* also results from a change in quiescence, irrespective of cell cycle length and/or numbers. With that in mind, our group has pioneered the concept that shortening G1 also changes the mode of division of NSC driving their generation of more NSC (reviewed in: Salomoni & Calegari, *Trends Cell Biol*, 2010; Borrell & Calegari, *Neurosci Res*, 2014). As a result, both cell cycle activity and number of cells increases, here by 6 and 2-fold, respectively, in line with our previous studies during development and adulthood (Lange et al., *Cell Stem Cell*, 2009; Artegiani et al., *J Exp Med*, 2011; Bragado Alonso

et al., *EMBO J,* 2019). To avoid confusion by the use of the generic term *"proliferative activity"*, we now adopted the more specific *"cell cycle length"* or *"cell number"* as appropriate.

Next, concerning percentage vs. number of cells, it should be noted that two contexts exist in which percentages, or alternatively total numbers, are preferable. On the one hand, when characterizing the cellular effect of 4D, indicating cell types as a percentage of targeted cells is preferable because some degree of variability exists in the injection and/or titer leading to slightly different pools of infected cells. Since 4D can only be expressed by GFP+ cells, representing values relative to GFP allows us to internally normalize for this effect. On the other hand, the reviewer is entirely correct in saying that for what hippocampal function is concerned, the absolute number of neurons, rather than their relative increase, is what matters. Hence, we now have provided both relative and absolute increases whenever meaningful and appropriate (e.g. new Fig. 1C-D, 2B, 4G).

To finally answer the reviewer's question (and as also requested by reviewer 2), the number of newborn neurons resulting from our manipulation are depicted in the new Fig. 1C and D and corresponding to about 30-70 neurons per mm2 of DG. We agree that this may seem negligible when considering the total number of all neurons, newborn plus mature, of the whole DG. This is in fact a remarkable aspect emerging from our study, that despite being seemingly small, this increase is still sufficient to trigger beneficial effects. We believe that this is due to the remarkable electrophysiological properties of these immature cells and their ability to amplify their influence through effects on mature granule cells, as extensively reported and discussed in our manuscript.

6. Page 4 bottom. "Acute 4D overexpression (together with GFPnls) in 16 months old mice for 3 weeks resulted in a 6-fold increase in overall proliferation and active NSC among targeted (GFP+) cells relative to controls infected with GFPnls viruses (EdU+: 0.77±0.22 vs. 0.16±0.11% and EdU+Sox2+: 0.53±0.14 vs. 0.08±0.07%; p=0.014 and p=0.008, respectively), which was paralleled by a doubling in NSC (Sox2+S100β–: 6.17±1.1 vs. 3.73±0.74%; p=0.033) (Fig. 1A)." Neither the figure legend nor the text is very clear in taking the less initiated reader through what is being shown here. The reference is to Figure 1A. The text says "increase in overall proliferation" – this is not explicitly referred to in the Figure. The text says things are bigger in GFP+ cells relative to controls with GFPnls viruses. The figure has different colour symbols for GFP and 4D groups, with GFP being in fact the control group The text says "paralleled by a doubling in NSC". But, again, neural stem cells are not referred to explicitly in the figure. So – while I do not doubt the assertions – the text is one thing and the figures to which the text refers are another, with one or both being written in "lab jargon" that is not explained to the reader. Finally, there is a classic example of looking at the data from two different premises in Figure 1D. The text states that the persistent neurogenesis is 4-fold higher in the older animals who have had a longer time in the chronic study ("… However, the absolute value of the ratio of DCX+ to GFP+ declines with age to a value of < 1% in the older animals. Thus, a reader is required to look at the declining absolute level of the ratio in Figure 1D to derive mentally that the relative ratio is increasing. This is OK (I guess) but hellishly confusing.

We apology for the confusion. We have corrected these inconsistencies in the revised manuscript. We believe that questions concerning *"proliferative activity"* and *"absolute vs. relative values"* have been addressed in our previous response (point 5). Considering our use of jargon, we now indicate in parentheses the characteristic marker(s) (e.g. NeuN+/BrdU+) used to identify a particular cell

type and/or effect (e.g. NSC, newborn neurons, cell cycle activity, etc) and do so whenever referring in the text to such population and using the same labels in the figures. To address the reviewer's last concern, a new graph depicting the evolution of relative increases in newborn neurons in 4D/GFP mice has been added (Fig. S1C) to help visualizing this effect.

7. Am I right in worrying that there is a confound between acute vs chronic treatment, and old vs young? This may not matter, but it is either unfortunate or the reasons for doing this not well explained. Perhaps it's fine.

Possible, we have now revised our manuscript entirely and checked that all references to acute vs chronic and young vs old are correct.

8. Page 8: "..and supports the notion that neurogenesis may play a role in memory consolidation (32)." The notion that neurogenesis plays a role in memory consolidation seems extremely unlikely, if only for timing reasons. If neurogenesis is triggered by a learning episode, the proliferation, integration and connectivity of the new neurons takes about a month. This is too long a time before the rate at which memory consolidation could be changed, as much of it would already be done, and thus hard to see how it could possibly have much impact. I guess greater sparsity of representations and more immature dentate neurons might affect the efficiency of memory consolidation guided by CA1 sharp waves, but this could not be in any way relevant to the information that actually triggered the neurogenesis. This idea is surely a non-starter except as a non-specific effect.

We regret the misunderstanding. While it has been proposed that neurogenesis might play a role in memory consolidation (e.g. Kitamura et al., 2014), we did not mean to imply that the neurons *"triggered by a learning episode"* are the ones responsible for consolidating the memory of such event. The reviewer is correct, times are different, a *"non-starter"*. It is only when neurogenesis is triggered prior to learning and for a period of time that is long enough for the newborn neurons to integrate that they may at this point participate in the learning and memory of a future event. This could occur through various effects in the circuit, including those discussed in our manuscript from changes in the excitation/inhibition balance to sharp-wave ripples in CA1.

Triggering neurogenesis without learning or other confounding effects is a key aspect and power of our 4D approach distinct from previous studies in which neurogenesis was increased by undefined, systemic and/or cognitive stimuli such as enriched environment or physical exercise. In our study, mice received either GFP or 4D viruses and neurogenesis increased genetically for a well-defined period of time prior to learning. Only after were the mice subjected to learning for the first time. Hence, in our study neurogenesis was never *"triggered by a learning episode"*. We apology for the lack of clarity and have now better described this aspect in our revised manuscript.

9. In this context, the data of Figure 2D are simply not credible. It is claimed, in both the title of the figure and the detailed text, that neurogenesis triggers a decrease in activity. A decrease is seen in cfos, but the electrophysiology shows no such effect with massive variability and overlap between the control and experimental conditions. The assertion that these data are significant at the p = 1

x 10 to the 10 level (eg. Fig 2D right) cannot be an accurate description of the data that is shown. Note also that the n numbers are simply too small for comfort with group sizes of N=3 to N=6. This is of major concern.

The box-whisker plots shown in Fig 2E represent quartile/outlier plots because the data are not normally distributed and, hence, non-parametric statistics were used resulting in the distribution of the data appearing much broader (quartiles instead of standard error of the mean). This has been clarified in the manuscript. In addition, this analysis was not based on a group size of 3 and 6 as stated by the reviewer. These numbers refer to the mice (N) used for the experiment while the group size for analyses was the number of ripples (n), well over 1,500 per condition. Statistical methods are now better described in our revised manuscript and together with the use of non-parametric analysis, we believe that this addresses the reviewer's concerns about the p-values calculated on thousands of data from different mice and resulting in a wide spread of quartiles/outliers but still reaching highly significant differences.

10. Page 10 "Consistent with the known effect of aging on navigation (7-9), young mice displayed a greater use of contextual (allocentric) relative to procedural (egocentric) strategies than old mice throughout both the learning and reversal phases (odds ratio young vs. old: contextual=1.85 and 2.28, procedural=0.34 and 0.48; Wald-test p=0.033 and 0.002; respectively; Fig. S3B and S3C)." Given the importance of the claim, the deposition of these poorly explained findings in the supplementary feels wrong. A further difficulty is the failure to explain with any clarity how the difference between navigational strategies is identified, measured or tested. A figure of what are claimed to be different strategies is shown in Figure 3 but it is unclear how actual paths are analysed with respect to these exemplars. Is a single path classified as one or another? Or might a strategy change half-way through a trial? The data analysis is very vague. To add that the data appear to show that the mice appear to be moving at between 4 and 6 cm/sec. This feels slow to me for mice in a typical open-field unless there is a lot of stopping – but again maybe it is OK.

We have now considerably expanded the description of this method. Summarising this here: we implemented the approach described by the Kempermann group (and others) for the Morris water maze (quite a few papers, e.g.: Garthe et al., 2009, 2013, 2014 and 2015) by combining it with a dry-land version of the task, the Barnes maze. The whole path of a single trial was assigned to a single strategy by 2 experimenters blind to the manipulation and following the adapted criteria mentioned above and in our revised manuscript. Finally, linear regression analysis was used to assess the data and odds ratios calculated to obtain p values. An entire methodology paper was published describing the statistics behind the whole method (Garthe et al., *Hippocampus*, 2015).

In relation to Fig. S3A comparing young vs. old, unmanipulated mice the reviewer states that: *"Given the importance of the claim, the deposition of these poorly explained findings in the supplementary feels wrong."* We partly disagree, the age-related switch from allocentric to egocentric learning is very well-described in the literature and so these data were needed essentially as a "positive control" for us to know that our strategy assessment, previously used only in young mice, also worked in old mice. Important to us, yes, but neither novel nor surprising to the general reader and for this reason sent to the supplementary information. The reviewer is correct that these mice are slow as they are old and usually slower than young mice.

12. Discussion: I find it difficult to follow the strand of the Discussion, with claims that (a) strategy choice rather than the efficacy of any one strategy may be what matters – though the data behind this claim are largely descriptive, and (b) that increasing NSC results in a change in sharp wave ripples which I have previously noted seems not to be shown in the results of Figure 2.

We rewrote the discussion entirely. Indeed, we believe that strategy choice can be more indicative of brain function than the performance of the strategy itself because the latter is relative and subjective depending on the specific conditions that are applied. For example, the reviewer can imagine a maze in which the target position is never changed. It is evident that a target-directed, allocentric strategy would here give the best performance. Conversely, if the target position is changed every day, adopting an allocentric strategy would no longer be effective and an egocentric strategy may now become the smartest choice. Any combination of target position, geometry of the maze and other parameters may make a novel strategy to superficially appear as *"the best"*. This was the case in our test in Fig. 3 where a reduced performance occurred in 4D, but not control, mice when the target location was changed. Yet 4D mice always kept a bias toward more complex allocentric navigation than controls. We invite the reviewer to also refer to our response to reviewer 2 point 10 about the (incorrect) alternative interpretation that 4D mice are unable to *"flexibly learn"*.

Concerning Fig. 2, we addressed the concerns about the analyses of ripples above. In addition, we would like to emphasize that several observations concur in this figure to consistently show a major role of neurogenesis in reducing the excitatory tone of the hippocampus. With the new analyses added to our revised study this includes: i) sparser activity in the DG as assessed by c-Fos immunohistochemistry, ii) direct contact between newborn neurons mossy fibres and parvalbumin inhibitory interneurons in the CA3, iii) increased activity of these interneurons in both the CA3 and iv) CA1 and, finally, v) a reduced occurrence, duration and internal frequencies of CA1 ripples. Together, we think that these data well fit several recent reports and considerably extend the current knowledge about the role of adult neurogenesis.

We hope to have now satisfactorily addressed the importance of strategy choice and the inhibitory effect of neurogenesis in an entirely revised discussion and thank the reviewer for giving us the opportunity to better dissect these important aspects.

REVIEWER #2 (REMARKS TO THE AUTHOR):

Berdugo-Vega and colleagues present an interesting set of findings exploring the contributions of enhanced and inducible adult neural stem cell (NSC) expression in the hippocampus in adult mice to behavior and physiology. Specifically, this study implemented virally-mediated Cdk4/cyclinD1 (4D) overexpression (Please use the term retrovirus-this is absent from the entire manuscript) to significantly enhance NSC expression in aged animals, but also in younger animals (e.g., in 7 months of age). 4D overexpression in the hippocampus resulted in changes in physiological readouts of the hippocampus function (e.g., alterations in CA1 LFP signals and behavior-induced c-fos expression in the dorsal hippocampus) while also altering behavioral strategies in aged mice seen in various navigational tasks. Aged mice normally become more dependent on procedural strategies (which may be a detriment to context-dependent behavior and result from loss of normal

hippocampal function), but aged mice treated with 4D appeared to rely more heavily on contextual cues after learning, suggesting that it may rescue some hippocampal function via facilitation of NSC induction. Moreover, improvements in context-dependent behaviors were observed even if chronic 4D treatment was applied in young mice that were later tested after aging (such effects may relate to interaction with activity in the striatum, at least based on c-fos analyses). Overall, this is an interesting study that attempts to link increases in adult hippocampal neurogenesis with circuit properties and behavior. However, the authors try to cover too much ground leaving out key comparisons and controls and making the interpretation of the data very difficult.

We thank the reviewer for his/her interest in our work and the many constructive comments that we tried to address in a thoroughly revised manuscript and point-by-point responses below.

Primary concerns:

1. What are the levels of NSCs and progenitors in these aged Nestin mice prior to 4D injections?

The levels of NSC prior to 4D injections, i.e. in unmanipulated mice, is well described in the literature and consistent with our assessment in mice injected with GFP, control vectors for which we used Dcx and birthdated NeuN+ cells as a read out of neurogenesis. It follows that the number of NSC should very much reflect these quantifications and, also in response to reviewer 1, we now provide these values also as absolute numbers in addition to relative increases.

2. The 4D Off design is hard to interpret with the information provided. This is because the 4D ON cells differentiate into adultborn neurons even in the absence of TAM (Many of the 4D expressing GFP cells appear to be in the GCL at 3-4 weeks post infection (Fig.1A) and so the net population of extra neurons (generated after TAM) are of many different ages. So the 8 week timepoint is likely to have neurons that are less than 8 weeks of age. Please discuss adequately.

As mentioned in our introductory statement and in response to reviewer 1, we appreciate the comments and regret our lack of clarity in properly describing the 4D-On/Off system and the use of lentiviruses, which we now addressed in a revised introduction, results and new Fig. 1.

The reviewer's comment about *"many GFP cells appearing in the GCL"* likely results from the assumption that we used regular retroviruses (also next point). This was not the case, we used lentiviruses targeting all cells, including mature neurons in the GCL. As described by our group in young mice, our system relies on the use of lentiviruses because retroviruses failed to achieve 4D-increased neurogenesis probably due to their targeting mainly of neuroblasts more than NSC proper (see in particular Fig. 4 in Artegiani et al., *J Exp Med*, 2011). Since lentiviruses also transduce any other cell in the DG, we designed a system to discriminate between infected mature neurons vs. NSC-derived, newborn neurons by using nestin-CreERt2 mice in which recombination in NSC triggers the removal of the nls of GFP (both in 4D as well as controls; see new Fig. 1A). Occurring within nestin+ cells, this leads to the generation of an age-matched cohort of newborn neurons that are birthdated and labelled by cytoplasmic, rather than nuclear, GFP. We suspect that the reviewer's comment probably referred to nuclear-GFP+, mature neurons in the GCL a population that is distinct from NSC-derived, cytoplasmic-GFP+, newborn cells (e.g. Fig S2). Importantly, 4D expression in mature neurons had no discernible effect on their morphology,

physiology, activity, etc. as shown by a number of studies from our group during development (Lange et al., *Cell Stem Cell*, 2009; Nonaka-Kinoshita et al., *EMBO J*, 2013), adult hippocampal (Artegiani et al., *J Exp Med*, 2011) and SVZ (Bragado Alonso et al., *EMBO J*, 2019) neurogenesis.

3. I am not convinced that the 4D OFF system reverts levels of neurogenesis to baseline. Because of small overlap between Nestin CreeRT2 recombined cells and activated stem cells and progenitors that are infected by the 4D retrovirus (at 3 or 4 weeks post infection of 4D virus), the frequency of CrERT2 deletion of NLS and 4D cassette is likely to be very low. This means that escaper 4D ON cells will continue to generate new neurons and will be very hard to detect since aged mice show extremely low levels of stem cell activation. Relatedly, do neural stem numbers return to baseline in 4D OFF experiments? Please show Nestin RGL GFP following TAM in 4D OFF condition.

Concerning the normal levels of neurogenesis after 4D OFF: We counted Sox2+S100β– NSC 2 weeks and Dcx+ cells 4 weeks after tamoxifen and in both cases found levels similar to control (see new Fig. S2). We hence conclude that after 4D OFF neurogenesis reverts to control levels.

In addition, we do not think that *"the frequency of CrERt2 deletion of NLS and 4D cassette is likely to be very low"*. To the contrary, we think that this is quite high. Considering our use of lentiviruses (not retroviruses) targeting all cells (i.e. quiescent and active NSC, progenitors, newborn and mature neurons) it is clear that only a tiny fraction within this population are nestin+ NSC. It is only within this subpopulation that recombination may occur. Within this pool, recombination is highly efficient, specific and escapers negligible as assessed when establishing our system testing different tamoxifen regimens and two nestin-CreeERt2 lines generated by the Eisch or Kageyama labs (we finally used the latter). For example, in Artegiani et al., 2011 the reviewer can see that the proportion of nestin+ among GFP+, infected cells (2.2% in controls and 5.7% after 4D expansion of NSC; Fig. 3E) is de facto identical to that of cells undergoing recombination and becoming cytoplasmic, GFP+ upon tamoxifen (2.3% in controls and 6.8% after 4D expansion of NSC; Fig. 5D). The reviewer can also see in the current study that quantifications of the 4D-effect as total number of cells per area and fold-increases have similar magnitudes (Fig. 1C) again consistent with a high recombination efficiency.

Finally, on a purely theoretical ground, it is not essential for our study that neurogenesis returned to baseline. In the chronic experiments neurogenesis is never returned to baseline and yet improvements in learning, navigation and memory were also found in this context.

4. Please provide data for 4 and 6 week YFP neurons rather than just DCX and Edu/NeuN.

These data are now shown in Fig. 1B-C and S1A-B.

5. Provide total DCX counts for Fig. 1B analysis. Total counts give a sense of how neurogenesis is changing in the hippocampus. If recombination efficiency is very low, then it is likely that there are very few newly added neurons. This is important information for the reader. The authors do discuss the very low numbers (approx. 100 extra DCX cells) in aged mice but this data (exact numbers) needs to be plotted and conveyed to reader.

We do agree and as also requested by reviewer 1, we have now included absolute numbers in addition to relative percentages. Particularly for newborn neurons, we refer to 4-week old, birthdated, NeuN+ cells as a more mature stage than Dcx.

6. Fig. 1C: Brdu/NeuN/GFP overlap: The NeuN signal within dotted line is not convincing.

We again agree and have replaced these pictures although the high density of granule cells in the DG makes it difficult to appreciate individual nuclei (if not at high magnifications for counting).

Fig. 1D. What is the effect of chronic overexpression for 3, 6, 9 months on size of NSC pool and total count of 4 week old neurons at each of these timepoints selected for behavior?

Regrettably, we did not perform these quantifications but we find it very reasonable to conclude that, based on the count of Dcx and NeuN neurons in Fig 2 and S2, the number of NSC and 4 week old neurons should very much reflect these very same magnitudes to the point that repeating these year-long, chronic experiments may not be justified.

7. I appreciate that the authors are trying to compare DG c-fos levels relative to CA3 and CA1 (as an indicator of relative excitation in the network) in Figure 2B, however, the mean values of CA3 and CA1 should also be reported. For example, although c-fos levels (per mm2) in DG appeared lower, that doesn't exclude the possibility that CA3 or CA1 c-fos levels are unchanged (relative to controls); moreover, the lower values of DG/CA1 divisions shown in Fig. 2B could actually be biased by higher levels of CA1 (or CA3) relative to controls, thereby generating lower values for DG/CA3 or DG/CA1 calculations. Similar claims can be made about the c-fos data shown in Figure 4G; reporting DG, DM, and DL values (in addition to the division analyses) will greatly aid in the interpretation of the relative impact of 4D induction on the network.

The reviewer is entirely correct. We have now abandoned the less informative relative counts and provided numbers per mm2 as suggested. We have done this not only in previous regions of the DG, CA3 and CA1 but also by restricting this to the more relevant suprapyramidal blade of the DG and sub-population of parvalbumin+, inhibitory interneurons of both CA3 and CA1.

In addition, we are particularly grateful for the reviewer's comment because this guided us to detect GFP+ mossy fibres contacting parvalbumin cells in the CA3 (see new Fig. S2B). Since these processes were detected by their cytoplasmic-GFP label, this revealed their origin as 4-week old, 4D-derived, newborn neurons highlighting the potential of newborn neurons to directly drive feed-forward inhibition to CA3. Intriguingly, our data fit well with a recent report by the Sahay lab in which a genetic increase in the connectivity of mossy fibres promoted memory precision in old mice (Guo et al., 2018), which would extend to newborn neurons a role that was previously thought to be restricted to mature granule cells (see revised discussion). Together, our previous and new data reinforce our conclusion about the effect of neurogenesis on DG sparsity and increased inhibitory tone of the hippocampus and thank the reviewer for the excellent suggestion.

8. Fig.2B. Essential comparisons (Young mice and learning naïve mice versus aged mice) are missing to support authors' claims. The authors must provide cfos data for aged and young learning- naïve mice. Also, it is simply not clear from the data that the cfos changes reflect "sparsity of memory representations". This claim needs to be addressed using memory tagging approaches or in vivo imaging of memory ensembles. This phrase should be should be removed from manuscript.

Together with the new quantifications of c-Fos described above, we have also performed the comparison of young and old, learning-naïve mice as requested by this reviewer. The data are intriguing in that we found a clear, minor but significant, general reduction in the counts of c-Fos+ cells per area in old, as opposed to young, mice (see reviewer-Fig 1 below). While a reduction of c-Fos in the DG of old mice was expected and explained by the known loss in afferent path connections during ageing, a reduction in CA3 was instead less expected and somehow puzzling. This is because a large body of literature, by using electrophysiological methods, has firmly established that ageing triggers hyperexcitability of the hippocampus and particularly so of the CA3. Yet, this known hyperexcitability of aged mice was not reflected by an increase, but rather a decrease, in c-Fos density (reviewer-Fig 1). This divergence between electrophysiological and immunohistochemical methods was similarly observed in our data showing changes in the electrophysiological tone of CA1 ripples in 4D-treated mice but without a corresponding detectable change in c-Fos counts (Fig 2). From this we concluded that c-Fos may be insufficient to address neurophysiological data such as firing patterns of the c-Fos+ labelled ensembles. We feel that a proper discussion of these data of young and naïve mice would deviate from the main message of our study that, as the reviewer stated, already *"tries to cover too much ground"*. Hence, we have included these data here for the reviewer but not in the revised manuscript.

Concerning memory tagging, this is an excellent suggestion and we started establishing these methods including rtta-cFos AAV and catFISH. These experiments will require ageing a significant number of mice and corresponding resources, both with regard to time and money, and therefore decided to perform these experiments in the context of a parallel project focussed on 4D manipulations in young mice that we plan to conclude within short time (see also response to reviewer 3, point 1 concerning this parallel study on young mice). Therefore, as suggested by the reviewer, we removed references to *"memory representations"* from the revised manuscript.

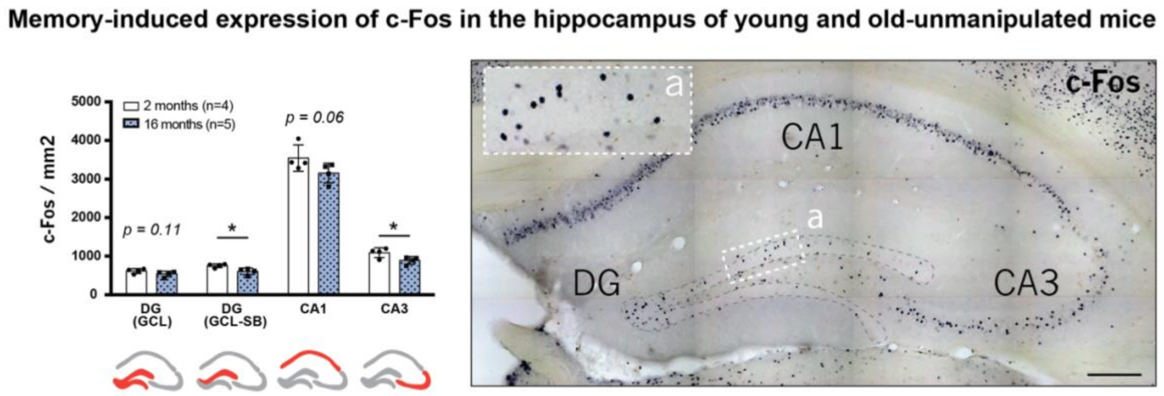

Figure depicting the age-related changes in c-Fos expression in the trisynaptic circuit upon fear memory expression in young vs. old unmanipulated mice.

9. I believe the authors need to clarify further what is meant by testing for electrophysiological recordings in animals during a “natural drowsy state” (equivalent time of day for the mice?, etc.). I appreciate that testing was done in a dimly lit conditions, but it is difficult for the reader to determine if differences in inter-ripple intervals (for example) can be attributed to the 4D treatment, or if there were potential differences in the levels of arousal at test that account for the findings in 4D vs. controls (i.e., perhaps the inter-ripple intervals were shorter in controls because they exhibited more of them during the session). Can the authors indicate whether 4D vs. controls differed in the total number of identified ripples for the recording sessions?

We significantly expanded the description of these experiments in the methods section and also indicated that, no, 4D mice did not display any major difference in arousal state with wakefulness and drowsiness being identified by an increase in theta-frequency activity during the former state and finally selecting for analyses a similar total number of ripples (>1,500) as now explained in methods (see also reply to reviewer 1, point 9 about ripple analyses).

10. Previous work has shown that amplitude and frequency of SWRs increase in CA1 after contextual fear conditioning. How does the authors manipulation affect SWRs following learning?

This is a good suggestion addressed in a parallel study from our group and now being prepared for publication. We refer the reviewer to our reply to reviewer 3, point 1 about this second work.

11. The authors observe a dissociation between behavioral measures and search strategies. This is interesting but requires greater discussion. For example, Fig. S3E and Fig. 3C argues against improved reversal learning as 4D mice show increased perseverance behavior. According to the latency plots, the controls do not exhibit increased latency when reversal begins. Previous studies have reported a congruence between search strategy and behavior. Please discuss further.

We agree about the need of a more detailed discussion that was lacking in our previous version. The differences mentioned by the reviewer are a critical aspect of our study. The reviewer correctly states that “*controls do not exhibit increased latency when reversal begins”.* The reason for this is clear in that old, control mice predominantly navigate randomly or by chaining in circles at the periphery of the maze (red and yellow; Fig. 3 and S3). These mice are simply unable to acquire any substantial use of place-directed, allocentric navigation (blue) and it is for this reason that control mice do not need to display flexible spatial learning upon reversal. They simply keep on moving randomly or by chaining, exactly as before, without a change in latency or pathlength because the performance of these egocentric strategies is independent from the target position.

Only mice that acquired place-directed, allocentric navigation (4D mice) are expected to show a decreased performance upon reversal. As a result, we think that it is not appropriate to conclude that a decreased performance is due to a *“lack in flexible learning”*. In fact, we found that perseverance (black) in 4D mice disappears after a few trials and, hence, that 4D mice are perfectly able to flexibly re-learn the new target position doing so, again, by allocentric navigation. Validating this, novel ANOVA analyses specifically during re-learning were added to our study.

In turn, this should deliver two important *"warning messages"* to the field: First, that assertions about *"flexible learning"* without a corresponding assessment of learning strategies can be seriously misplaced. Second, that a decreased behavioural performance (latency) is not necessarily due to cognitive impairment but, conversely, may result from the use of more complex and cognitively demanding learning strategies (allocentric). We hope to have better addressed these concepts in our revised manuscript and thank the reviewer for raising these points.

11. Fig. S4E-F: n=4-5 mice per group is not sufficient for meaningful interpretation of behavior especially, when there is also variability related to viral injections and TAM mediated deletion etc…

The n in this graph does not represent mice but group of days with A-B or B-A order. For these experiments >10 mice were used (N) with group of days (n) being compared.

12. Do the authors see an effect of 4D expansion on forgetting?

To the contrary, we see better retention! This is an additional important aspect of our study and, as the reviewer probably knows, potentially challenging current hypotheses. Particularly in Fig. 4, both in the case of fear conditioning and navigation, probe trials at weeks or even months after training yielded a better retention as well as a better re-acquisition of memory in 4D mice. We are however hesitant to make a strong point out of these observations and imply that neurogenesis is not involved in forgetting, which might still be the case, because in our study what control and 4D mice have to recall are "*different types of memory*" formed during the use of "*different learning strategies*". It might simply be that one type of memory is easier to retain than the other, we don't know, but different types of experiments can address this, which we are pursuing in an independent study focussing on young mice (see reply above about SWR after learning and our response to reviewer 3, point 1 below).

Other concerns:
1) Figure 2A reports n's of 5-6 for freezing behavior (which are noted as the basis for Figure 2B), but the graphs in Figure 2B only show 4 animals per group. Can the authors clarify/fix?

This has been clarified in the figure legend for Fig. 2

2) In the main text and figures, the authors list significant p values, but without showing the exact F (or t) values for these tests or their degrees of freedom (for example) that led to that p value. I recognize the methods state that tests were used as appropriate, but without showing such information, the reader must infer any main effects or interactions, and thus cannot tell if post-hoc comparisons are used appropriately.

Values were added as appropriate.

3) 4D induction depends on successful incorporation of the virus in the DG. There is no mention of whether animals were or were not excluded from experiments based on successful "hits" or "misses" of viral injections. I assume the authors have done their due diligence in this regard, but it should be stated if and how the authors identified successful 4D or GFP infection in their experiments.

This is correct, and the information added to the methods. Essentially "missed hits" were so rare (<10%) and evident that we didn't need a criterion to define them.

4) c-fos is stated as counts from granule cell layer of DG; is this from the dorsal, ventral, or both blades? Noting such locations may also be important because representative images in Figure 1 (for the DCX quantifications) appear to show cells from the medial DG (with both blades) but other images just show a single blade, so it's not clear where quantifications are being made or if we should have any expectations of differences in these counts at various medial-lateral segments of the DG. Also, if possible, and to better understand how representative these counts may be, it may benefit readers to know the size (expressed as mm2, for example) of the domain quantified for each subregion.

Areas considered are now explained in the methods and depicted as small drawings below each figure. Briefly, neurogenesis was quantified in an entire series of DG sections, including dorsal and ventral hippocampus. Exceptionally, NSC were quantified only in the SGZ of those hippocampal series (as indicated). C-Fos was quantified exclusively in the dorsal hippocampus.

5) In Figure 1B and 1D, is there a reason we are only shown DCX+/DAPI+ in the images, given that the authors are showing data comparisons for DCX+/GFP+ percentages? Also, the yellow tick marks are very small, and not easily discernable unless the reader zooms in quite a bit. I doubt it will show up well in a printed version, I would recommend making the images larger, and/or making yellow arrows larger.

We made the pictures and arrows bigger. GFP channel was excluded from the DCX sections because it made the difference in Dcx number easier to appreciate.

6) I will leave this up to the discretion of the authors, but it's unclear to me why box-and-whisker plots are used for some data presentations while mean/SEM (though sometimes they are stated as SD) (with individual data points) are shown for others. Certainly, the some of the behavioral data could be represented with means/SD/SEM, thus allowing reader to see individual performance in these tasks via individual data points.

We agree that the inconsistencies are unfortunate. However, the non-normal distributions seen in our physiological metrics make box-whiskers plots necessary even if such representation cannot be applied in certain conditions, such as cellular quantifications, that are based on fewer datapoints. Given this, we used box-whiskers plots whenever N>5 and mean/SD in cellular analyses. Mean/SEM was preferred for latencies and pathlengths to better appreciate the mean evolution of performances throughout the test. These different representations are now explained in the methods, legends and throughout the text whenever a new test is introduced.

7) The use of "old people" in the text may be construed as offensive; I recommend changing.

We now use the neutral *"during ageing"* and without referring to *"people"*.

REVIEWER #3 (REMARKS TO THE AUTHOR):

- The authors claim that the expansion of neurogenesis counteracts the effects of aging on several aspects related to hippocampal functioning. However, only old animals have been used in most of the analyses shown. Thus, it is not possible to conclude that this manipulation counteracts the effects of aging unless the effects were tested on young mice too. In this regard, cfos+ cell counts, electrophysiological recordings, and behavioral test should be performed on both young and aged mice (GFP and 4D groups), in order to correctly interpret the effects of 4D overexpression on aging. Otherwise, no conclusions regarding the effects of 4D overexpression on the changes triggered by aging can be reached. If authors do not wish to consider performing these experiments, the whole manuscript should be re-focused. E.g., referring to rejuvenation navigating strategies would not be possible, given that the control effects of 4D on young animals is unknown.

We generally agree that comparing young mice is important and have now analysed young-control mice including for c-Fos (see reply to reviewer 2, point 8) and ripples (below). But before discussing these data, we first would like to explain why including young-4D mice in the current study would not be appropriate. There are several reasons for this including, above all, the need to use different behavioural tests for old and young mice. Moreover, testing for cognitive gains requires challenging tasks because easy ones in which control already reach *"nearly maximal performance"* are, by definition, unlikely or impossible to be further improved. The reviewer can see an example of this in our recent study on olfaction in which 4D-mice showed improved discrimination exclusively when addressing a very difficult, but not easy, task (Bragado Alonso et al., *EMBO J*, 2019). The same effect can be seen in our current study in tests performed throughout life related to which we wrote (page 15): *"an increase in neurogenesis was associated with differential behavioural gains depending on the difficulty of the task and the age of the animals"*. This is the reason why the field overwhelmingly uses the more challenging Morris water maze for young mice while using the Barnes, dry maze for old ones.

With that in mind, we did perform a parallel study, including of navigation, of young-4D mice using the Morris water maze and other tests and again found improvements upon increased neurogenesis. These improvements are, however, of a rather different nature relative to those of old mice because young-control mice barely use egocentric navigation and easily reach *"nearly maximal allocentric performance"*. Hence, this aspect could not be *"further improved"*. An example of this is shown in our study in which control mice at 14 months (certainly not young) still retained a significant degree of allocentric navigation that was lost by the 18th month (compare GFP in Fig. 4 and 3). While our second study of young mice is being prepared for publication, we believe that the many differences between tests used, phenotypes observed and cognitive processes involved make them unsuitable for a merge, particularly so considering the criticism of reviewer 2 about our study already trying to *"cover too much ground"*.

Concerning the requested quantifications of c-Fos and ripple analysis in young, naïve mice, we refer the reviewer to our response to reviewer 2, point 8 concerning the former and show below the results concerning the latter. Again, differences were observed between the 3 groups (young-control, old-control and old-4D) but we are unable to meaningfully interpret these differences. A large literature already exists that describes the many effects of ageing on the brain including, but not limited to, its cellular, neurophysiological, systemic and behavioural effects. Differences are in fact so many and profound that an entire field is dedicated to understand their significance. This prevents us from making strong conclusions about these differences and, hence, we do agree with the reviewer that our lack of knowledge prevents us from making claims about our approach *"counteracting the neurophysiological effects of ageing"*. We nonetheless believe that claims about *"counteracting the behavioural effects of ageing"* can still be made when referring to egocentric vs. allocentric and procedural vs. contextual learning and memory as, in fact, these hippocampal-specific cognitive behaviours were made functionally closer to that of young mice. We have therefore revised our manuscript accordingly to remove any claim directly or indirectly linked to *"neurophysiological effects"* and thank the reviewer for spotting our mistake.

[redacted]

- The expression of cfos should be analyzed in naïve animals not subjected to behavioral tests. Moreover, absolute numbers of cfos+ cells for every anatomical region should be provided in all cases.

Please see new data in Fig. 2 and Fig. S2 of the revised manuscript as well as our response to reviewer 2, above. We now have analysed c-Fos accordingly in different anatomical regions as proposed and, additionally, assessed activation within the subpopulation of parvalbumin, inhibitory interneurons of both the CA3 and CA1. Notably, these analyses allowed us to identify direct contacts between mossy fibres belonging to newborn neurons and parvalbumin inhibitory interneurons in the CA3. Since these processes were detected by their cytoplasmic-GFP, this

revealed their origin as 4-week old, 4D-derived, newborn neurons highlighting the potential of these cells to directly drive feed-forward inhibition to CA3. This is a novel aspect of our study, reinforcing the role of 4D-increase in neurogenesis in reducing the overall hippocampal activity.

- This Reviewer believes that there are several inconsistencies regarding the statistical analyses performed. For example, was normality determined throughout the manuscript? Which statistical test was used? Were all the variables tested normal? Why was SD represented in cell counts whereas SD was used to represent behavioral analyses? Moreover, repeated measures ANOVA tests should be used for behavioral analyses that are performed on the same mouse over consecutive days.

We have thoroughly revised the entire manuscript and better described our statistical analyses in results, methods and figure legends. In particular, whenever a new test was used in accordance with the type of distribution of the data, this was indicated the first time in the text together with the respective values. Unfortunately, inconsistencies remain in depicting the graphs, which is due to the very different N and/or n used in each group of experiments (e.g. N=3-4 for cellular analyses, 10-12 for behavioural ones and >1,500 for ripples). We favoured, and used throughout, SDs with the only exception of behavioural performances in Fig. 3C where their use led to a very confusing representation due to major overlaps in SD error bars throughout all points. This distinction is indicated in the figure legend.

Minor points
ABSTRACT
- In the last sentence of the Abstract, the authors affirm that the results were obtained “by exploiting the brain’s endogenous reserve of neural stem cells”. However, this Reviewer believes that, given that a transgenic mouse + viral injections have been used, referring to the endogenous reserve of stem cells is not totally appropriate in this context.

By *“endogenous”* we imply that these NSC are not being added by, for example, transplantation but physiologically reside within the brain. We believe that this is very important because many initiatives worldwide aim to use exogenous cells, i.e. ESC, iPSC, NSC, etc for neurodegenerative diseases. We show that these exogenous cells may not be essential in all cases.

INTRODUCTION
- In addition to feedfodward inhibition to mature neurons, the authors may wish to cite other aspects of newborn neuron behavior that are important for hippocampal functioning.
- In page 3, authors affirm that “In contrast to behavioral performance, effects of neurogenesis on learning strategy choice were never explored”. They migh wish to cite Garthe et al. 2009, PMID 19421325.
- The concept of hippocampal-estriatal memory competition has not being correctly introduced. Please expand its description and relevance for the present work.
- Also, the mouse model that is going to be used should be described in the Introduction section.

We agree on all points, have made the changes suggested and added the citation indicated (Garthe et al., 2009 and 2015 are cited but not in the context of ageing since these studies are on young

mice). Also in response to the other reviewers' points, we included nearly 20 new citations and the recent observation of lateral inhibition within the DG by the Hen's lab (Luna et al., 2019).

MATERIAL AND METHODS

- In Fig S1, the number of cells counted per mouse should be indicated in the Figure legend.
- The type of viruses (lenti, AAV, ...) that have been used should be specified.
- Methods to perform cell counts should be described into much more detail.

The material and methods section has been considerably expanded and total counts added.

RESULTS

- The images shown in Figure 1 D are of poor quality. Please provide better images at low and high magnification showing the increase in the number of DCX+ cells.
- In Fig 2, the total numbers of cfos+ cells for the CA1 and CA3 fields should be provided. In Figure 2, all the graphs should be adapted to the same format.
- In S3 E, are these data obtained from old or young mice?.
- In Fig 4 G, images are of poor quality. It seems that almost all the neurons of the hemi-brain are positive for cfos. Authors should provide more convincing images of the cells that are being quantified. Moreover, the nomenclature used to define striatal areas is not very standard. Are they analyzing the caudate/putamen/GP structures? If so, how were these regions anatomically identified?
- Examples of cfos+ cells are provided both in Fig 2 and 4. However, immunofluorescence or immunohistochemistry images are shown in Figure 4 and 2 respectively. Were different methods used to quantify these number of cells in different experiments? Do the authors consider that this could affect the number of cells detected? As previously mentioned, absolute number of cells should be provided instead of cell ratios.

All these changes were made. Please refer to the revised manuscript for each. Fig. S3D-E referred to GFP and 4D-treated old animals. We have expanded the methods to better describe the identification of striatal areas. Concerning the use of different methods to reveal c-Fos, yes, we initially used DAB and immunofluorescence and the reviewer's criticism is well to the point since we realized that caveats arise in putting data from the two methods together. We now focused our analysis entirely on immunohistochemistry and performed novel quantifications to fill up the gaps that arose for statistics.

DISCUSSION AND INTERPRETATION OF THE DATA

- In page 19, the affirmation that the increase in neurogenesis was performed "without systemic effects or interfering with the physiology of the niche or the neurons themselves" seems to be too heavy. These factors have not being analyzed in such depth in the present manuscript.
- In the same page, authors affirm that "key downstream effects on circuitry and balance with the striatal navigational memory system" have been identified. This affirmation is not correct, given that the hierarchy between these circuits has not been determined.
- The striatum is a complex structure, which participates in several circuits involved in memory. This Reviewer consider that the examination of cfos+ cells in two areas of this structure (which,

as indicated in the Major points, should be much more precisely described) is not sufficient for reaching this conclusion.
- Please consider that stereotaxic surgeries are being performed when affirming that “our study is the first to alter this competition without surgical or pharmacological manipulations”.
- The final sentence of the Discussion seems to be out of place.

We have removed, revised and/or toned down the statements pointed out here.

Reviewers' comments:

Reviewer #1 (Remarks to the Author):

This revised manuscript is accompanied by a courteous and detailed "rebuttal" letter from the authors that answers the concerns of the reviewers very well. They have been attentive to serious concerns about the methodology, statistics, and interpretation of the results, notably the issue of the extent to which this genetic manipulation of stem cells has influenced the absolute number of dentate granule cells that would then have an impact on inhibition and further processing in the Hippocampal formation. This number appears to be samll but they offer groups to believe that their impact could be considerable.

Moreover, while I still see the absolute numbers of mice used in the study as small, I commend the authors for adding additional experiments to address my own and the other reviewers' concerns.

The bottom line is that the novel use of this genetic 4D manipulation of stem cells, without the use of any kind of surgical or other invasive technique, has had the effect of positively altering the capacity of the dentate gyrus to separate patterns that, mediated by enhanced inhibition, increases the likely use of allocentric behavioural strategies in spatial navigation. This is a great improvement on studies using only fear conditioning for which this kind of information is unavailable.

Accordingly, I am now in a position to recommend acceptance of the manuscript.

Reviewer #2 (Remarks to the Author):

Berdugo et al

The authors have addressed many concerns but some key concerns are still not addressed.

1. Sox2 labels stem cells and progenitors. If the authors are claiming stem cell expansion in aging mice, please quantify Nestin+ Sox2 radial glial like cells. It is possible that the 4D manipulation drives progenitor expansion in aging (see Pilz 2018 Science). Sox2+ S100- is not sufficient to justify the claim of neural stem expansion.
2. The quantification of numbers of 4 week and 6 week old cytoplasmic GFP neurons is still absent. The authors say that they have done it in Fig. 1B-C and S1A-B but I do not see it.
3. The link between changes in neurogenesis, ripples and "reducing hyperexcitability" is not clear. Several groups have shown CA1 hypoexcitability and not hyperexcitability in aging and these papers are not cited nor discussed in light of findings (See work by Disterhoft lab). How does the analysis of ripples done here relate to "increased hypoexcitability" in aging is not clear. Inclusion of young mice (exp vs controls) is key to helping understand how changes in neurogenesis affect ripples. Comparison of young vs old CA1 (non-manipulated) will help reader understand age-related changes in ripples in CA1.

Reviewer #3 (Remarks to the Author):

The authors satisfactorily address most of my questions. I found the activation of parvalbumin interneurons in the CA3 a specially significant and exciting addition to the paper. I also find the new behavioral data on young mice has strengthened significantly the conclusions previously drawn. However, there are a few points that I believe require further clarification:
- There is a new paragraph that has resulted somehow confusing to me. It is the following: "Notably, this relative increase in neurogenesis among the pool of GFP+, infected cells was also confirmed by a

similar increase in the total number of 4-week old neurons (EdU+NeuN+) per area of the DG and irrespective of whether or not these neurons were derived from GFP+, infected NSC (4D vs. GFP: 34±6 vs. 9±4/mm2, p=0.005; Fig. 1C, right). This in turn suggested the highly efficient targeting of NSC and recombination by our floxed-4D viral approach". Why does it demonstrate the efficient targeting of NSC? I apologize if I missed something important, but I find these statements really unclear.
- I would still be interested in seeing the total number of cfos+ cells in Figure 4G rather than only the ratios between the DG and striatum. I think both the total numbers and this ratio should appear in the final version of the manuscript.

Remaining remarks
Reviewer #2

1. Sox2 labels stem cells and progenitors. If the authors are claiming stem cell expansion in aging mice, please quantify Nestin+ Sox2 radial glial like cells. It is possible that the 4D manipulation drives progenitor expansion in aging (see Pilz 2018 Science). Sox2+ S100- is not sufficient to justify the claim of neural stem expansion.

We agree that Sox2+ S100- may alone be insufficient to identify neural stem cells. Our claim is not only based on this evidence but also on previous quantifications made, indeed, on Nestin+ Sox2+ as well as GFAP+ and other markers (see: Artegiani et al. 2011). Moreover, with regard to *"radial morphology"*, it is unclear whether or not and to which extent such radial morphology is maintained in old age (e.g.: Bonaguidi et al., 2012; Encinas et al., 2012) and, to the best of our knowledge, whether or not the presence of non-radial NSC can be excluded.

2. The quantification of numbers of 4 week and 6 week old cytoplasmic GFP neurons is still absent. The authors say that they have done it in Fig. 1B-C and S1A-B but I do not see it.

If the reviewer refers to *"absolute numbers"* of cytoplasmic-GFP+ cells, providing these values would not be very valuable due to subtle differences in infectivity and viral titers across experiments. We believe that a better quantification of the increased levels of neurogenesis was provided after the first round of revision and following the suggestions of reviewer 1 and 2 by giving both relative and absolute numbers of EdU-birthdated/NeuN+ cells.

3. The link between changes in neurogenesis, ripples and "reducing hyperexcitability" is not clear. Several groups have shown CA1 hypoexcitability and not hyperexcitability in aging and these papers are not cited nor discussed in light of findings (See work by Disterhoft lab). How does the analysis of ripples done here relate to "increased hypoexcitability" in aging is not clear. Inclusion of young mice (exp vs controls) is key to helping understand how changes in neurogenesis affect ripples. Comparison of young vs old CA1 (non-manipulated) will help reader understand age-related changes in ripples in CA1.

The reviewer is correct that hyper-excitability during ageing was reported in CA3 pyramidal cells whereas CA1 has been reported to display hypo-excitability. Hence, that our reference to hyper-excitability was incorrect when generally referring to the whole hippocampus. We therefore limited our claims about reduced hyper-excitability to the CA3 specifically as supported by our finding of higher recruitment of inhibition in this area (Pv+/c-Fos+ cells) (Fig. 2D). Being CA3 a key generator of ripple activity in CA1 (Nakashiba et al., 2009), our observation fits well with our next finding of reduced ripple activity in this latter region (Fig. 2E). All references to CA3 vs CA1 hypo-/hyper-activity were now corrected throughout the manuscript (red text) and thank the reviewer for pointing out our incorrect statement.

With regard to ripple activity of young mice, and as we attempted to explain in the previous round of revision, interpreting difference between young and old mice would not only be very difficult but also distracting from the message of the current study which is certainly not about ripple physiology during ageing. Assessing the effect of neurogenesis in hippocampal activity (including ripples) and function

specifically in young mice is the focus on another study from our lab that is being prepared for publication. We therefore feel that ripples data of young mice would better fit in this new study.

Reviewer #3 (Remarks to the Author):

1) There is a new paragraph that has resulted somehow confusing to me. It is the following: "Notably, this relative increase in neurogenesis among the pool of GFP+, infected cells was also confirmed by a similar increase in the total number of 4-week old neurons (EdU+NeuN+) per area of the DG and irrespective of whether or not these neurons were derived from GFP+, infected NSC (4D vs. GFP: 34±6 vs. 9±4/mm2, p=0.005; Fig. 1C, right). This in turn suggested the highly efficient targeting of NSC and recombination by our floxed-4D viral approach". Why does it demonstrate the efficient targeting of NSC? I apologize if I missed something important, but I find these statements really unclear.

To be fair, the reviewer is correct that these numbers are not a *"formal demonstration"* that recombination must be high. As that sentence says: these data *"suggest"* that recombination is high. In fact, by sheer probability, it would be unlikely that both the relative as well as absolute increases would have the very same magnitudes if recombination should only affect a small fraction of all cells. Even less likely that the same *"coincidence"* occurred in our previous study (Artegiani et al., 2011) by using entirely different markers. Finally, since this reviewer was particularly interested in *"recombination efficiency"* also in the previous round of review, we would like to point out that even if the efficiency of recombination was low (which we believe was not), essentially nothing would change of our main conclusions and findings.

2) I would still be interested in seeing the total number of cfos+ cells in Figure 4G rather than only the ratios between the DG and striatum. I think both the total numbers and this ratio should appear in the final version of the manuscript.

We agreed with the reviewer about this in the previous round of review. Number of c-Fos+ cells in the DG and striatal areas were already added in Fig. S4.

REVIEWERS' COMMENTS:

Reviewer #2 (Remarks to the Author):

Round 2
The authors should not make claims not supported by the data. Yes, the Artegiani paper shows Nestin+GFP+ cell expansion but this paper does not. Here, It is only indirectly guessed to be the case. The authors agree that Sox2+S100- is not sufficient to identify stem cells. So please change the language to convey what the experimental design captures and cite the Ategiani paper to justify the inference. By not using the correct language to characterize data, readers and trainees can be confused and find claims to be misleading.

If in aging (we do not know) radial glial morphology cells are not maintained, then the authors manipulation must be expanding progenitors, no? Again, the authors need to convey these points clearly.

"1. Sox2 labels stem cells and progenitors. If the authors are claiming stem cell expansion in aging mice, please quantify Nestin+ Sox2 radial glial like cells. It is possible that the 4D manipulation drives progenitor expansion in aging (see Pilz 2018 Science). Sox2+ S100- is not sufficient to justify the claim of neural stem expansion.
We agree that Sox2+ S100- may alone be insufficient to identify neural stem cells. Our claim is not only based on this evidence but also on previous quantifications made, indeed, on Nestin+ Sox2+ as well as GFAP+ and other markers (see: Artegiani et al. 2011). Moreover, with regard to "radial morphology", it is unclear whether or not and to which extent such radial morphology is maintained in old age (e.g.: Bonaguidi et al., 2012; Encinas et al., 2012) and, to the best of our knowledge, whether or not the presence of non-radial NSC can be excluded. "

It is very important for the authors to provide direct evidence for their claim for expansion of 6 week old neurons. Why? Because it captures the population that are progeny of the original pool (stem cells and progenitors) that underwent recombination. Also see Reviewer #3 (comment #1). DCX and Edu/NeuN does not substitute for the GFP analysis. In the first round, the authors said that they did the analysis that I requested but they did not. Why is this important? One situation could be that increasing progenitors indirectly increasing neuronal survival of immature neurons by producing secreted factors. In this case, one may not see an increase in 6 week old GFP neurons because many non-GFP immature neurons will survive in response to secredted factor. In this situation, a low recomb efficiency in stem cells would result in survival of a larger fraction of immature neurons.

"2. The quantification of numbers of 4 week and 6 week old cytoplasmic GFP neurons is still absent. The authors say that they have done it in Fig. 1B-C and S1A-B but I do not see it.
If the reviewer refers to "absolute numbers" of cytoplasmic-GFP+ cells, providing these values would not be very valuable due to subtle differences in infectivity and viral titers across experiments. We believe that a better quantification of the increased levels of neurogenesis was provided after the first round of revision and following the suggestions of reviewer 1 and 2 by giving both relative and absolute numbers of EdU-birthdated/NeuN+ cells."

Comments #3.

OK.

Reviwer2 comments:

Reviewer #2 (Remarks to the Author):

The authors should not make claims not supported by the data. Yes, the Artegiani paper shows Nestin+GFP+ cell expansion but this paper does not. Here, It is only indirectly guessed to be the case. The authors agree that Sox2+S100- is not sufficient to identify stem cells. So please change the language to convey what the experimental design captures and cite the Ategiani paper to justify the inference. By not using the correct language to characterize data, readers and trainees can be confused and find claims to be misleading. If in aging (we do not know) radial glial morphology cells are not maintained, then the authors manipulation must be expanding progenitors, no? Again, the authors need to convey these points clearly.

We try to address this point from all possible angles.

First, available knowledge: We agree with the reviewer that a single marker X, such as Sox2 (or even nestin for that matter!), alone is insufficient to characterize stemness. More rigorously, the field uses a panel of markers in combination, as well as morphology, to increase the confidence about stem cell identity. This is exactly what we have done in our previous 2 studies upon 4D manipulation in the 2 neurogenic niches of the hippocampus (Artegiani et al., J Exp Med, 2011) and subventricular zone (Bragado et al., EMBO J, 2019) of young mice and ultimately demonstrating expansion of "neural stem cells". While not repeating all these quantifications in the current study, we still find it entirely appropriate to infer from the existing literature and knowledge that 4D can expand neural stem cells also in old mice for the simple reason that no rationale exists to assume that the effect of 4D should change during ageing.

Second, technical limitations: The problem in repeating the previous assessments done in young mice in the current study with the use of multiple markers and morphology is not only the significant costs and time of raising such animals (2 years old!) but also the fact that nestin (as a cytoplasmic antigen) is tremendously difficult (hence unreliable) to quantify at this age due to a high immunogenicity background notoriously associated with old tissues. This justifies our use of Sox2 (a nuclear antigen!). Even more so, and as appreciated by the reviewer

him/herself, it is completely unknown whether or not, and to which extent, neural stem cells retain radial morphology in the old brain. Given this lack of information about morphology, the unreliability of makers and the burden and time needed for these quantifications, we consider it unreasonable to request these experiments for the current study.

Third, fundamental definition: We would like to remind the reviewer that a "stem cell" is more fundamentally defined by 2 cell biological properties, rather than the expression of marker X and Y or shape. The 2 criteria that are both necessary and sufficient to fulfill are 1) unlimited self-renewal and 2) the ability to generate differentiated cells. Showing that 4D increases self-renewal in the hippocampal stem cell niche as well as the generation of differentiated neurons over the entire course of life of a mouse and up to 20 months of age fulfills, by definition, these 2 criteria even if (hypothetically) marker X or Y should no longer be expressed or detectable.

Fourth, relevance for this study: Even ignoring the above points, lets us assume that 4D in the old brain indeed only expanded progenitors but not stem cells. At least based on the definition of stem cell above, this would necessarily imply that 4D can "reprogram" progenitors into becoming functional stem cells… Beside the extraordinary finding of these new Yamanaka-like factors, the main conclusion and novelty of our study would not change the slightest. Still, increasing neurogenesis rejuvenated hippocampal function.

Fifth, semantic: The reviewer requests us to *"change the language"* and *"cite the Artegiani paper"* not to *"mislead the trainees"*. We are happy to comply and cite the Artigiani paper even more often than the current 9 times. In addition, when introducing the abbreviation "neural stem cells (NSC)" for the first time, we could specify that these are instead "neural stem and/or progenitor cells (NS/PC)" but doubt that this would significantly improve the quality of our work and be any less misleading, if not perhaps more, to trainees.

In conclusion, we feel that the requested assessment of "stemness" for the current study may appear technically unreasonable, conceptually unnecessary and ultimately irrelevant.
Yet, we agree with the reviewer that the use of Sox2 is alone not an indicator of stemnees and fully acknowledge the fact that potential differential effects of 4D on stem versus progenitor

cells were not dissected here. Therefore, we have now changed the text referring to the targeted cells as to include both "neural stem and progenitor cells (NSC)" and specified that our inference about a stem cell-specific effect is derived from our previous studies during embryonic developoment and adulthood (4 citations).

It is very important for the authors to provide direct evidence for their claim for expansion of 6 week old neurons. Why? Because it captures the population that are progeny of the original pool (stem cells and progenitors) that underwent recombination. Also see Reviewer #3 (comment #1). DCX and Edu/NeuN does not substitute for the GFP analysis. In the first round, the authors said that they did the analysis that I requested but they did not. Why is this important? One situation could be that increasing progenitors indirectly increasing neuronal survival of immature neurons by producing secreted factors. In this case, one may not see an increase in 6 week old GFP neurons because many non-GFP immature neurons will survive in response to secredted factor. In this situation, a low recomb efficiency in stem cells would result in survival of a larger fraction of immature neurons.

We do not understand this point. We never made any claim about *"increased 6-week old neurons"*. The only point in our study when we mentioned 6-week old neurons is when assessing synaptic density (Fig. S1). We made this choice because synaptic contacts are more clearly defined at this age, hence showing a normal morphological maturation. The only functionally relevant time point for our study is 4-week old neurons, not 6. This is because several studies have demonstrated that 4-week old neurons not only i) are transiently characterized by distinct electrophysiological properties than more mature neurons but also that ii) they have passed the critical period for survival and permanent integration in the hippocampal circuitry (key literature cited in our manuscript). It is for this very reason that behavioural tests were performed 4 weeks post tamoxifen, not 6. The reviewer also mentioned potential *"secreted factors"* leading to *"cell-extrinsic effects"* and triggering an hypothetical *"increased neuronal survival"*. Supposedly, these effects are ultimately dependent on a *"low recombination efficiency"*. Again, we fail to understand what rationale or evidence does the reviewer bring to assume that any of these effects should exist. To the contrary, we provided and discussed multiple evidence about both the absence of cell extrinsic effects and high recombination efficiency in Artegiani et al., 2011, literature from

other groups using the same nestin-Cre mouse line, our current work and our previous point-by-point response (particularly for high recombination efficiency, this very point was raised by reviewer #3 who was fully satisfied and convinced by our reply and data). As a final note, and similarly to the previous discussion about the definition of “stemness”, even if any of the effects speculated by this reviewer should exist, our main conclusion and fundamental novelty of our study that increasing neurogenesis rejuvenated hippocampal function would not change the slightest.
